# Computational Strategies for a System-Level Understanding of Metabolism

**DOI:** 10.3390/metabo4041034

**Published:** 2014-11-24

**Authors:** Paolo Cazzaniga, Chiara Damiani, Daniela Besozzi, Riccardo Colombo, Marco S. Nobile, Daniela Gaglio, Dario Pescini, Sara Molinari, Giancarlo Mauri, Lilia Alberghina, Marco Vanoni

**Affiliations:** 1 SYSBIO Centre of Systems Biology, Piazza della Scienza 2, 20126 Milano, Italy; E-Mails: paolo.cazzaniga@unibg.it (P.C.); chiara.damiani@unimib.it (C.D.); besozzi@di.unimi.it (D.B.); riccardo.colombo@disco.unimib.it (R.C.); nobile@disco.unimib.it (M.S.N.) daniela.gaglio@ibfm.cnr.it (D.G.); dario.pescini@unimib.it (D.P.); mauri@disco.unimib.it (G.M.); lilia.alberghina@unimib.it (L.A.); 2 Dipartimento di Scienze Umane e Sociali, Università degli Studi di Bergamo, Piazzale S. Agostino 2, 24129 Bergamo, Italy; 3 Istituto di Analisi dei Sistemi ed Informatica “Antonio Ruberti”, Consiglio Nazionale delle Ricerche, Via dei Taurini 19, 00185 Roma, Italy; 4 Dipartimento di Informatica, Sistemistica e Comunicazione, Università degli Studi di Milano-Bicocca, Viale Sarca 336, 20126 Milano, Italy; 5 Dipartimento di Informatica, Università degli Studi di Milano, Via Comelico 39, 20135 Milano, Italy; 6 Istituto di Bioimmagini e Fisiologia Molecolare, Consiglio Nazionale delle Ricerche, Via F.lli Cervi 93, 20090 Segrate (MI), Italy; 7 Dipartimento di Statistica e Metodi Quantitativi, Università degli Studi di Milano-Bicocca, Via Bicocca degli Arcimboldi 8, 20126 Milano, Italy; 8 Dipartimento di Biotecnologie e Bioscienze, Università degli Studi di Milano-Bicocca, Piazza della Scienza 2, 20126 Milano, Italy; sara.molinari.sm@gmail.com (S.M.)

**Keywords:** metabolism, metabolome, modeling, systems biology, genome-wide model, constraint-based model, core model, mechanistic model, ensemble modeling, parameter estimation, reverse engineering, flux balance analysis, network analysis, sensitivity analysis, control theory

## Abstract

Cell metabolism is the biochemical machinery that provides energy and building blocks to sustain life. Understanding its fine regulation is of pivotal relevance in several fields, from metabolic engineering applications to the treatment of metabolic disorders and cancer. Sophisticated computational approaches are needed to unravel the complexity of metabolism. To this aim, a plethora of methods have been developed, yet it is generally hard to identify which computational strategy is most suited for the investigation of a specific aspect of metabolism. This review provides an up-to-date description of the computational methods available for the analysis of metabolic pathways, discussing their main advantages and drawbacks.  In particular, attention is devoted to the identification of the appropriate scale and level of accuracy in the reconstruction of metabolic networks, and to the inference of model structure and parameters, especially when dealing with a shortage of experimental measurements. The choice of the proper computational methods to derive *in silico* data is then addressed, including topological analyses, constraint-based modeling and simulation of the system dynamics. A description of some computational approaches to gain new biological knowledge or to formulate hypotheses is finally provided.

## 1. Introduction

Metabolism can be viewed as a dynamic chemical engine that converts available raw materials into energy, as well as into the building blocks needed to produce biological structures and sustain multiple cellular functions. The hydrolysis of large substrates, such as complex sugars or lipids, into smaller molecules produces energy (*catabolism*), whereas *anabolic* processes go the other way round by synthesizing large complex molecules at the expense of ATP and often of reducing power. Catabolism and anabolism are highly interconnected and partially overlapping, forming a complex network of biochemical transformations. Moreover, they are coordinated by an elaborate regulatory structure to make the cell promptly responsive to a variety of chemical, environmental, genetic and developmental perturbations.

The number of low molecular weight metabolic intermediates and end products (*i.e.*, with a molecular mass smaller than 800)—collectively referred to as *metabolites*—has been estimated to vary from 274 in the simplest bacterium *M. genitalium* [1] to 2043 in *E. coli* [2], from 1458 in budding yeast [3] to 5063 in human [4]. The full complement of metabolites present in a given cell type within a given environmental, genetic and/or physiopathological condition, is referred to as the *metabolome* [5]. In turn, the metabolome can be divided into the *endo*-metabolome, referring to metabolites present inside the cell, and the *exo*-metabolome, referring to metabolites excreted by the cell or present in the extracellular fluids [6].

Metabolomics, the youngest of the *omics* technologies, is able to concurrently identify thousands of metabolites, which are generated by the enzymatic reactions of specific metabolic pathways. Considering that the different amounts of metabolites obtained under perturbed experimental conditions reflect the changes in enzyme activity, metabolomics allows to obtain a biochemical snapshot of the physiological and pathological state of a cell or an organism [7,8,9,10]. Metabolic profiling [11] provides a complete functional picture of the biochemistry that connects the genome—via transcription and translation—to a particular phenotype through the interaction between the cell and the environment [12]. For this reason, metabolomic applications have recently found a valuable use in clinical field [12], to identify new biomarkers in neurological, cardiovascular and cancer diseases [13,14,15,16,17,18]. At the same time, researchers in both academic and industrial fields have pushed forward metabolic studies in order to efficiently engineer microbial strains for the production of bulk and fine chemicals [19,20,21].

For these and other purposes, a system-level perspective on all metabolic interactions, typical of Systems Biology, is indeed beneficial to complement the classical reductionist approach of molecular biology. The focus of Systems Biology is to understand the regulation of cellular and intercellular processes in quantitative and predictable ways, thriving on the integration of methods and data that are typical of different disciplines, as Biology, Chemistry, Computer Science, Mathematics, Physics, *etc.* [22]. According to the Systems Biology paradigm, the biological system of interest needs to be formally described with a mathematical model. Two key features of modeling are the possibility to formulate *in vivo*-testable hypotheses and to integrate different experimental data, especially those measured with high-throughput techniques as transcriptomics, proteomics and metabolomics. In this context, there is a growing need for models able to analyze the regulatory features of metabolism, as well as to give structure and predictive power to post-genomic data.

Metabolism can be described with mathematical models defined at different levels of detail, ranging from *genome-wide models*, which include several thousands of reactions and metabolites, to *toy models*, that consider only a few reactions, passing through *core models*, usually characterized by hundreds of reactions and metabolites.

**Figure 1:**
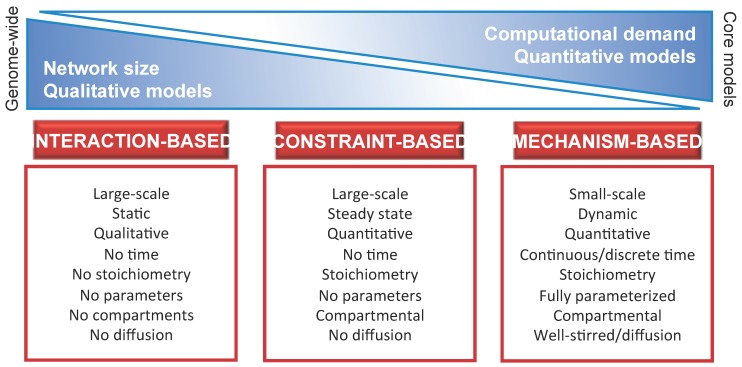
Schematic overview of the main modeling approaches for biological systems, together with their principal characteristics and differences. Moving from the coarse-grained (*interaction-based*, *constraint-based*) to the fine-grained (*mechanism-based*) approach, models vary with respect to: (i) the *size* of the system, defined in terms of the number of components and respective interactions included in the model, which decrease from genome-wide to core models (Section 2.1); (ii) the *computational costs* required for the analysis of the model, which increase from the analysis of the topological properties of the network typical of interaction-based models (Section 3.1), to the study of flux distributions typical of constraint-based models (Section 3.2), to the investigation of the system dynamics typical of mechanism-based models (Section 3.3); (iii) the nature of the computational results together with the predictive capability, which changes from *qualitative* to *quantitative* while moving from interaction-based models (characterized by a high level of abstraction) to mechanism-based models (fully parameterized and describing the system at the level of the functional chemical interactions).
- Choose the most appropriate *mathematical formalism*. A one-to-one correspondence between each modeling approach and a specific modeling purpose would facilitate the choice of the most suitable strategy to be employed. Unfortunately, a sharp-cutting separation is not always possible. In general, mechanism-based (dynamical) models—which are usually defined as systems of differential equations—are considered the most likely candidates to achieve a detailed comprehension of cellular processes. Nonetheless, the usual lack of quantitative parameters represents a limit to a wide applicability of this approach for large metabolic networks. Various attempts have been proposed for the automatic estimation of missing parameters or the characterization of the parameters space [24,25,26].
  On the other side of the spectrum of modeling approaches, interaction-based models are characterized by a simplified representation of the biological process and allow to achieve qualitative knowledge only. These models can be analyzed by using, for instance, graph theory or topological analysis to investigate the “design principles” of metabolic networks, that can be considered transversal to different organisms [27]. Moreover, they allow to easily identify the so-called *hubs* (highly interconnected components, essential for the existence of several metabolic processes), as well as the metabolites and reactions connecting them, which can be of particular interest within the scope of, e.g., drug target discovery [28].
  Considering the limitations of these modeling approaches, the common practice for the computational investigation of metabolism usually relies on constraint-based models. These models are based on the definition and manipulation of stoichiometric matrices, whose native application pertains to the field of metabolic engineering. In this case, the methodologies that were initially developed for the optimization of microbial strains or for the maximization of some product yields in biotechnological applications, are now widely used with different goals in the study of metabolic networks.

The scale of the model is closely related to the choice of the proper modeling and computational approach, which is constrained by two key features: the nature of the biological system under examination, and the experimental data that are available or that might be measured for that system. More precisely, the modeler need to consider the following issues:

- Establish from the beginning the *scientific question* that motivates the development of the model. Consequently, the analysis of the model is expected to increase the current knowledge on the system, thanks to novel predictions on its functioning and to their experimental validation. In this phase, initial experimental data are necessary to define a plausible mathematical model, since they can aid to discriminate among different hypotheses on the structure of the system.
- Identify the proper *level of abstraction * necessary to formally describe the components of thesystem and their mutual interactions. In particular, the model should take into account all available knowledge on the biochemical, physical or regulatory properties of all system components and interactions. In so doing, any detectable emergent property of the system—either in the physiological state or in response to genetic, chemical or environmental perturbations—can be discovered with the appropriate computational methods. The choice of the level of abstraction will bring to the definition of either fine-grained (e.g., *mechanism-based*) or coarse-grained (e.g., *interaction-based* or *constraint-based*) models. Typically, the mechanism-based approach deals with toy or core models, while the interaction-based and constraint-based approaches are more suited for the analysis of genome-wide or core models. A schematic overview of the three main modeling approaches is given in Figure 1, including a list of their principal dichotomic features [23], such as quantitative *vs.* qualitative, static *vs.* dynamic, parameterized *vs.* non parameterized, single volume *vs.* compartmental, well-stirred *vs.* heterogeneous (diffusion), *etc.*

**Figure 2:**
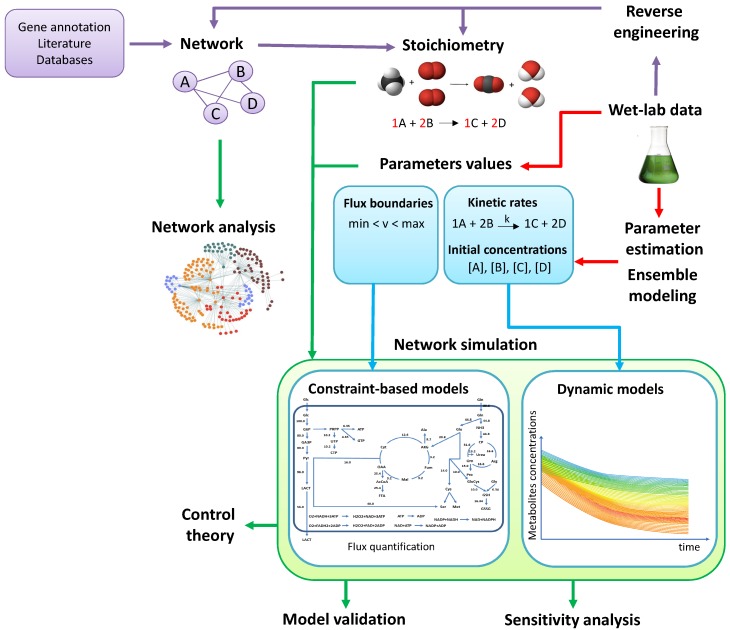
General scheme of the computational investigation of metabolism, from network reconstruction to *in silico* analysis. Violet arrows indicate the relationships between experimental data and reverse engineering methods used to reconstruct the metabolic network and to identify the stoichiometry of the reactions (see Section 2.1 and Section 2.3) Red arrows indicate the relationships between experimental data and the methods to derive or estimate the unknown parameters (see Section 2.2 and Section 2.4). Green arrows indicate the computational analyses that can be performed on metabolic networks and models (see Section 3 and Section 4). Blue arrows indicate specific computational analyses that can be carried out on different types of models (see, in particular, Section 3.2 and Section 3.3).

The aim of this review is to provide a global picture of the different modeling approaches and their related computational strategies for the analysis of metabolism. We will give indications on the limits and strengths of the various methods, and present several examples on the way they have been effectively applied with different goals. However, considering the very broad scope of this work, some research on metabolic modeling had to be necessarily omitted. The general workflow of the topics discussed in this review, which range from the reconstruction of metabolic networks to their *in silico* analysis, is presented in Figure 2. Accordingly, the paper is structured as follows. In Section 2 we provide an overview of the approaches to reconstruct metabolic networks at different scales, from genome-wide to core models, along with some computational strategies based on parameter estimation, reverse engineering and ensemble modeling, that are needed to automatically derive the metabolic network and the corresponding parameterization. In Section 3 we present some computational methods to derive *in silico* data from metabolic networks and dynamic models, such as topological analysis and Flux Balance Analysis for large-scale metabolic networks, and simulation methods for small-scale metabolic networks. In Section 4 we describe some strategies suitable for model validation and for an in-depth investigation of metabolism, such as sensitivity analysis and control theory. In Section 5 we discuss some relevant papers that show how different metabolic problems can be addressed with different modeling approaches. Finally, in Section 6 we conclude with some notes and future perspectives on this research field.

## 2. From Experimental Data to Models

Metabolism can be described by means of mathematical models defined at different levels of detail. The scale of the model concerns both the size of the biological system that one has to analyze, defined in terms of the number of metabolites and reactions, and the chosen level of granularity. Except for very small metabolic networks, model development entails an appropriate choice of which metabolites and reactions need to be part of the model, in a tug-of-war between the desire to include as many molecular details as possible, and the opposite need to simplify the structure of the model.

### 2.1. Metabolic Network Reconstruction

The reconstruction of the network of metabolites and reactions in a given cell represents the starting point to develop a computational model of metabolism [29] (see Figure 2). In this context, a metabolic network can be represented as a graph, consisting of a number of components, called *nodes* (representing metabolites), and their respective mutual relationships, called *edges* (representing metabolic reactions). These formal representations are typically created in a bottom-up fashion based on genomic and bibliomic data, which can possibly be integrated with data obtained from laboratory experiments (as discussed in Appendix). Network reconstructions can vary in size (from genome-wide networks to smaller models focusing on specific metabolic pathways), and can be characterized by different levels of abstraction, according to the scope of their formulation. In this section we illustrate some noteworthy examples of genome-wide, core and toy models of metabolism, putting emphasis on the computational strategies used for the reconstruction of the network, as well as on the strengths and weaknesses of these approaches. The generation of networks derived from top-down approaches (inference of component interactions based on high-throughput data) will be instead discussed in Section 2.3.

The focus of this section is essentially on the process of reconstruction of the network, which represents the first step for further (either quantitative or qualitative) computational analyses. In what follows, we mainly provide examples of network reconstructions that are intended for quantitative analysis, e.g., constraint-based modeling or algorithms for the simulation of the dynamics (as described in Section 3.2 and Section 3.3, respectively). Nevertheless, it should be mentioned that the starting point for the development of the genome-wide models mentioned hereby are, often, metabolic network reconstructions that are intended to summarize the available experimental knowledge and may be studied with qualitative methods (e.g., the topological analysis described in Section 3.1). These manually curated maps are collected in various databases, such as EcoCyc [30], HinCyc [31] and KEGG [32].

**Genome-wide models.** One of the main purposes in the reconstruction of genome-wide (GW) models is to summarize all the current knowledge concerning metabolic processes at the level of single gene annotation, trying to take into account every single reaction that is known to occur within an organism. These reconstructions can be exploited in a dual way: on the one hand, they represent the scaffolds for different computational analyses, such as those described in the next sections; on the other hand, they work as repositories of all the collected knowledge about metabolic pathways [33].

The curation of GW models became possible after the development of high-throughput technologies [34], able to generate an unprecedented wealth of quantitative data about living systems [35]. In particular, omics sciences provided the main contribution (genomics, transcriptomics, proteomics, metabolomics, *etc.*), by giving a general view upon the parts that compose a specific class of compounds in the cell (DNA, RNA, proteins, metabolites, *etc.*), thus allowing the identification of all the components of the functional networks. GW models therefore represent the result of the integration of different kinds of information about metabolism. The first GW metabolic model was developed in 1999 for the bacterium *H. influenzae* [36]. Thanks to their great potential to provide a better understanding of the complexity of metabolism, several reconstructions appeared in literature during the last decade, covering a variety of organisms from bacteria [37] to lower [3,38] and higher eukaryotes [4,39], including plants [40]. These models, especially those of simple organisms like bacteria or lower eukaryotes, are often defined starting from previous versions of the same metabolic model [41], which then undergo iterative cycles of analysis, refinement, expansion and validation.

Because of their extension and complexity, GW metabolic network reconstructions are often the results of a community effort in which researchers are coordinated with a jamboree-approach, which may require many years of work [42]. This meeting-based reconstruction can indeed facilitate the achievement of a unanimous result, referred to as “consensus models” (see [4,43] for some examples). As extensively described in [42], in order to speed up this procedure, automated strategies of metabolic network reconstruction are typically exploited to aid the creation of an initial draft model of a selected organism, starting from its genome sequence or annotations, or from an existing model of a related organism [44]. Nevertheless, the process of automated reconstruction presents several limitations; for instance, it does not provide information about growth conditions or biomass composition. Moreover, the initial gene annotation could be incomplete or not always accurate, requiring an additional process of model curation and refinement. This stage is mainly based on data integration and manual control of the initial draft model, but it can also be supported by semi-automated procedures, especially for what concerns the process of gap-filling—needed to identify missing reactions in the network—for which many tools exist [45,46,47,48,49].

An open challenge in Systems Biology is the reconstruction of a reliable model of human metabolism, which would allow the analysis and in-depth studies of several diseases linked to metabolic disorders. The relevance and complexity of this challenge is assessed by the presence of many databases, whose goal is to build the ultimate human metabolic pathway, and by the efforts spent to reconcile them [50,51].

In this context, the most updated and complete human GW model reconstruction is, at present, *Recon 2* [4]. Given that human metabolism is much more extended than that of simpler organisms and hence more laborious to curate, the bottom-up multi-step strategy used for its reconstruction is a good representative of the general guidelines for GW metabolic network reconstruction, and will be therefore concisely illustrated hereby.

Recon 2 was curated starting from a previous version of the global human metabolic network, *Recon 1* [39], a reconstruction defined by exploiting an accurate human genome sequence annotation, which allowed to identify the initial set of metabolic genes. From these genes, by taking into account the protein-gene relationships, it was possible to identify the metabolic enzymes and hence the reactions that they catalyze (the edges of the network). These reactions were carefully formulated considering the stoichiometry of reactants and products, the substrate specificity, the directionality and reversibility and, altogether, they account for the overall conservation of mass and charge-based metabolite ionization. Moreover, metabolites in Recon 1 were correctly compartmentalized to properly consider transport and exchange reactions in the model. The entire reconstruction process consisted of various rounds of refinement and validation, and it was supported by literature data analysis which included online databases, primary articles, reviews and textbooks. Finally, the development of Recon 2 as an extension of Recon 1 was achieved by adding metabolic information from extra sources following, again, a step-wise strategy. Metabolic pathways already present in Recon 1 were extended, while other pathways were added *de novo*, resulting in a total number of 7440 reactions, which is almost doubled with respect to its previous version.

It is worth mentioning here that the specific metabolic pathways that actually operate within a cell are regulated by the interaction between the genome and the environment. Therefore, from a single genomic-data driven reconstruction, many condition-specific models can arise. For instance, generic GW reconstructions of human metabolism (such as Recon 2) have been used for the generation of cell type-specific models [4,52], according to the information about different cell proteomes contained in the Human Protein Atlas [53].

Despite the high level of detail, which makes them a good repository of metabolic information, GW reconstructions can present several limitations when one wants to simulate and analyze their behavior using the computational methods described in Section 3. First of all, since a GW network generally involves thousands of metabolites and reactions, the simulation of its dynamics becomes impracticable, not only because of the lack of knowledge on the kinetic parameters, but also for the computational costs it would entail (see Section 3.3). For this reason, GW modeling typically exploits Flux Balance Analysis (FBA) as reference computational method (see Section 3.2). However, the presence of inconsistencies in the network—mainly concerning mass and charge balance, and/or the lack of reactions or metabolites that are currently unknown—often prevent the description of the correct metabolic fluxes within the network [54]. For instance, the existence of thermodynamically infeasible loops should always be verified when performing computational analysis. To solve this issue, some *ad hoc* versions of FBA have been developed (see, for instance, [55] and [56]). Furthermore, the complexity of the network may represent an obstacle for a proper interpretation of the outcomes, resulting from the interaction of thousands of metabolites and reactions. In spite of these potential limitations, several successful applications of GW models have been reported [41,57,58,59].

**Toy and core models.** An opposite strategy to the reconstruction of GW models is the definition of models with a simple structure and including a limited number of components, which are often referred to as *toy models*. The goal of these models is not to describe in molecular terms a specific pathway, but rather to highlight some major regulatory properties. In a toy model, many pathways and thousands of reactions are thus summarized in a few steps, in order to more easily identify the most relevant components of the system. Good examples can be found in [60,61], where toy models were defined to probe the relationship between the type of energetic metabolism (respirative *vs*. fermentative) and cellular growth.

**Figure 3:**
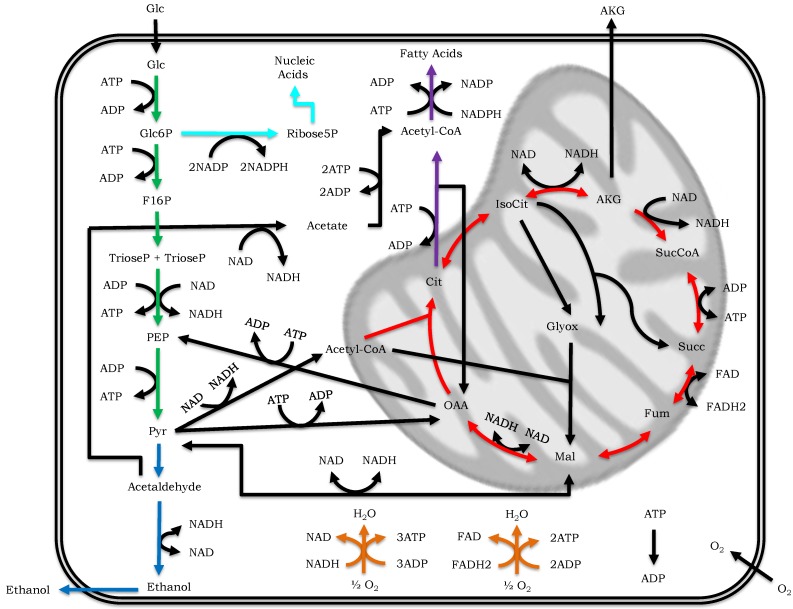
Example of core model representing the main metabolic pathways of yeast (modified from [65]). The pathways included in this example are: glycolysis (green arrows), ethanol fermentative pathway (blue arrows), pentose phosphate pathway (light blue arrows), fatty acids biosynthesis (violet arrows), tricarboxylic acid (TCA) cycle (red arrows) and oxidative phosphorylation pathway (orange arrows).

Core models (CMs) stay in between toy and GW models. They can cover in molecular details only one or a few simple pathways (e.g., glycolysis) [62,63], or else they can summarize information about several pathways, by including only those elements that are deemed to be essential to understand the regulatory features and/or the dynamic behavior of the phenomenon under study (see Figure 3 for an example).

A representative of the former type of CMs is the metabolic reconstruction presented in [64], which takes into account the glycolysis and the pentose phosphate pathways only. It is worth noticing that these models are suitable to be extended and/or plugged into a more complex model, including other pathways.

An example of the latter type of CMs is instead provided in [65], where the aim of the study was the identification of the design principles behind the Crabtree effect (a metabolic behavior that is characteristic of several yeasts [66]). In [65] the authors developed a CM including the main pathways that are known to play a role in the emergence of the Crabtree effect but, in order to both reduce the model complexity and avoid cell-type specificities, reactions that belong to a linear cascade without branching were lumped together in a unique fictitious reaction. Another example is the constraint-based model presented in [67], which takes into account the metabolic pathways that are supposed to have a pivotal role in cancer cell growth, namely, glycolysis, TCA cycle, pentose phosphate, glutaminolysis and oxidative phosphorylation.

CM and toy models make the analysis and interpretation of simulation results easier, but over-simplification may lead to erroneously neglect fundamental multi-factorial relationships between the state of the system and its response to different perturbations (e.g., in the modeling of complex diseases) [68]. On the other side, as the size of the model increases (in terms of number of metabolites and reactions), so do the parameters describing, e.g., the kinetic constants and initial concentrations of the chemical species: hence simulation results may be flawed by poor constraining of parameters by biological data, which are often qualitative or semi-quantitative at best. For this reason, CM and toy models may results very effective either to investigate very specific aspects of metabolism or to outline complex phenomena [69].

### 2.2. Parameter Estimation

When modeling metabolic networks, or any generic biochemical system, one of the main computational problem that has to be faced consists in the definition of all model parameters, such as kinetic constants or initial molecular amounts. A proper model parameterization of the model is an indispensable requisite to perform dynamical simulations, in particular for the analysis of CMs or toy models. In a few cases, the unknown parameters can be obtained by means of laboratory experiments but, in general, they are hard or even impossible to measure, especially if one aims to determine the *in vivo* values of kinetic rates. This experimental limitation has led to the development of computational methodologies for parameter estimation (PE) [70], whose goal is the automatic inference of model parameters (see Figure 2).

In order to face the PE problem, two fundamental requirements have to be satisfied. First, we need a precise knowledge of the physicochemical interactions between all molecular species involved in the system (*i.e.*, the network of reactions). Second, target time series are necessary to carry out the automatic inference of the unknown parameters. To this aim, the more precise and densely sampled the measurements are, the more reliable will be the inference of the model parameterization. In particular, time series of molecular amounts of some pivotal species, possibly obtained in different experimental conditions, can largely facilitate the assessment of the quality of the inferred set of kinetic parameters [71,72,73]. The model parameters can be determined by means of optimization techniques [70], whereby the simulated dynamics of the system is compared to experimental data, or by probabilistic estimation techniques [74], in which a posterior probability distribution of parameter values is assessed. In addition, a good practice consists also in coupling PE with computational methods for parameter identifiability [75] which, given the target data, allow to evaluate how accurately the value of each parameter can be estimated.

In the context of metabolism, a PE approach was proposed in [76] to estimate the kinetic parameters of seven different models of the biosynthesis of valine and leucine in *Corynebacterium glutaminicum*. These models, constructed using the information from KEGG [32] and MetaCyc [77] databases, were based either on Ordinary Differential Equations (ODEs) or Stochastic Differential Equations (SDEs) (see Section 3.3). To lessen the complexity of the PE problem, some of these models were simplified to avoid reversible reactions. So doing, the model parameterization was reduced from 59 down to 18 parameters. However, the downside of this strategy is that the simplified models were no longer able to correctly fit the laboratory observations. Indeed, most of the simulated dynamics—especially in the case of models with irreversible reactions—tend to become straight lines, thus behaving in a biologically implausible manner.

Another example of application of a PE method was presented in [78] for a detailed model of metabolism and energetics in skeletal muscle, relying on *in vivo* data. In this work, the authors proposed a multi-step, large-scale estimation of parameters which involves information from fluxes and concentrations dynamics. The model parameters were estimated by using a generalized reduced gradient algorithm [79], a method designed for the optimization of large-scale systems, that incorporates some constraints on resting, steady state and flux-concentration relationships. In order to reduce the search space of the large number of unknown parameters (90), the authors also presented a method to analytically compute some of them. In addition, sensitivity analysis methods (see Section 4.2) were exploited to assess the influence of the variation of parameter values on the metabolic responses during ischemia. Similarly, in [80] 42 out of a total of 359 parameters of a large model of the electron transport system of mitochondria, the oxidative phosphorylation, the Na^+^/Ca^2+^ cycle and the K^+^ cycle, were estimated exploiting a Monte Carlo optimization algorithm based on simulated annealing (SiAn) [81], and refined using a gradient-based method.

This kind of methods, also called local search algorithms, are unlikely to yield a proper set of physiological parameters, since their results strongly depend on the starting point of the optimization process (*i.e.*, the initial guess of parameters). To avoid this drawbacks, multi-start approaches should be rather exploited in order to collect a set of putative model parameterizations.

In [76], the authors compared the performance of a large set of PE techniques, showing that two optimization methods—Particle Swarm Optimization (PSO) [82] and Differential Evolution (DE) [83]—can yield significantly better results with respect to other techniques, as previously suggested in [84], where PSO and Genetic Algorithms (GAs) were compared. PSO, DE and GAs are population-based meta-heuristics, that is, strategies that exploit a population of candidate solutions (*i.e.*, model parameterizations), which can be recombined during the optimization process. These methods can yield, in general, better results compared to local search algorithms; however, they require a fine tuning of specific algorithmic settings, which allow to control the exploration of the search space of all model parameterizations. Indeed, PSO and DE can outperform all other algorithms only when these settings are properly identified. In [76] the authors also showed that Tribes [85] algorithm—a settings-free version of PSO—represents a good PE technique. In particular, this algorithm is suitable even in the lack of expertise on optimization methods or on the fine tuning of other PE techniques.

A simpler methodology for PE was proposed in [86] to the purpose of defining a general approach for metabolic engineering. To carry out PE, the authors exploited an ODE-based model consisting in a hypothetical branched metabolic pathway that leads to the production of two specific metabolites. Authors analyzed various global optimization strategies (GAs, SiAn) and gradient descent techniques (Levenberg-Marquardt [87]), putting evidence on the fact that the former can avoid local minima and allow a better exploration of the search space. This example highlights that, as mentioned above, the gradient-based methodologies should be avoided for PE, because of the non-linearity and multi-modality of the search space of model parameterizations.

Concerning the PE of large-scale models, in [88] the Cooperative Enhanced Scatter Search method was applied to different models of metabolism, requiring the estimation of hundreds of parameters. This method exploits the concept of cooperative parallelism to overcome the typical difficulties of PE: the high non-linearity of the problem, the large number of parameters to be estimated and the scarcity of experimental data that can be generally used as target of the optimization.

It is worth mentioning that one of the main limitations of all existing PE methodologies is that they are computationally very demanding, especially in the case of models consisting in high numbers of molecular species and chemical reactions. Notwithstanding this, the calculation of the goodness of different model parameterizations can be parallelized using any parallel architecture, such as clusters of machines or Graphics Processing Units (GPUs) [71].

### 2.3. Reverse Engineering

The methods for PE described in Section 2.2 are useful to fill the gap of missing kinetic data of a given network reconstruction. These reconstructions, as widely discussed in Section 2.1, are typically defined thanks to human expertise, by relying on pre-existing knowledge and on the available experimental data of cellular processes, as well as by exploiting various databases containing information on molecular interactions and pathways. However, in some cases the cascade of spatio-temporal interactions and of molecular mechanisms occurring in living cells might be not clearly established yet. This lack of knowledge solicited the development of automatic reverse engineering (RE) methods, to devise a plausible network of biochemical reactions that is able to reproduce the experimental observations (see Figure 2).

The RE problem may embed the PE problem when a dynamical model needs to be inferred, since in this case both the network topology and the corresponding set of parameters have to be optimized. Therefore, as in the case of PE, RE methods require the availability of target time series of the molecular species involved in the system, so that the quality of the reverse engineered models can be evaluated.

In general, the computational complexity of RE methods grows at least quadratically with the size of the biological system under investigation [73]; therefore, RE methods are usually applied to infer CMs characterized by a small number of molecular species and biochemical reactions. In the context of metabolic pathways, an application of RE methods was proposed in [89], where an evolutionary technique called Genetic Programming (GP) [90] was exploited to reconstruct the process of ketogenesis. GP-based approaches allow the simultaneous evolution of a set of candidate networks of reactions and their corresponding kinetic parameters, and have been frequently exploited for similar tasks, like the RE of models based on ODEs [91,92]. The ketogenesis pathway was also used as a reference case study in [93], which presented a two-level evolutionary algorithm, based both on a variant of GP (called Cartesian Genetic Programming [94]) for network inference and on PSO [71,82] for the respective PE.

A major drawback of RE methods is that, although they are able to generate mathematical models whose simulated dynamics can perfectly fit the experimental target data, they suffer the issue of *indistinguishability*, a problem that occurs when different network topologies exhibit the same dynamical behavior [95]. As a consequence, RE methods usually require an additional screening of the inferred models based on human expertise, as well as a validation phase to assess their (biological) plausibility.

If simple interaction networks are to be inferred, not requiring the estimation of kinetic parameters, then correlation-based RE methods [96], relying only on steady state data, can be successfully applied. In such a case, some perturbations of the system (e.g., variation of metabolites concentration) can be used to calculate correlation coefficients and entropy-based mutual information values, which are exploited to build a putative interaction network (*i.e.*, two metabolites are connected by an edge in the graph describing the network if their correlation is high). Then, the network is simplified and pruned by identifying and removing indirect interactions. Arkin *et al*. proposed a modified approach of correlation-based RE relying on time series data, which were used to calculate time-lagged correlations between metabolites [97]. The final network topology was obtained by applying an arbitrary threshold to remove the edges with lower correlation. Time-lagged correlation was exploited also in [98], coupled with probabilistic model calibration, to the aim of automatically identifying and removing false positive edges. The latter are determined by calculating those reactions which have an unlikely, or null, kinetic constant. They also provide examples of more sophisticated alternative approaches relying on transfer entropy [99], calculated between couples of time series of the chemical species. The authors compared the feasibility and limitations (e.g., the emergence of false positive and negative edges) of these methodologies on the metabolic pathway of Gemcitabine.

A way to detect indirect interactions and to perform the RE of a metabolic pathway whose edges in the interaction network have a specific *directionality*, consists in analyzing the concentration profile of metabolites, as proposed in [100]. This method works by increasing the concentration of a metabolite and analyzing its impact on the time series of other metabolites. In [100] the feasibility of this RE method was shown by reconstructing the glycolytic pathway.

Even though RE methodologies provide scientists with mathematically sound methods for the creation or the extension of biochemical models, they are currently limited by the following intrinsic issues: (i) the aforementioned indistinguishability problem; (ii) when using correlation-based methods, the optimization strategies can be ineffective (e.g., they can yield wrong edges in the interaction graph), especially if one has to deal with noisy target data [73]; (iii) the high computation time albeit, as in the case of PE, the computation of the quality of the candidate solutions can be parallelized, thus reducing the running time required.

### 2.4. Ensemble Modeling

Ensemble modeling (EM) is an approach suitable for the reconstruction of mechanistic models of metabolism [101], whose aim is the investigation of the model behavior under different perturbations (see Figure 2). EM consists in a large set of candidate models, based on elementary reactions characterized by mass-action kinetics, that achieve a certain steady state flux distribution in a given experimental condition. This strategy permits to capture the behavior of enzymatic reactions, in the case of complete knowledge of reference fluxes, enzymes and metabolites concentrations in the whole network. The kinetic parameters of the ensemble models are randomly chosen according to two main criteria: (i) they must be thermodynamically compliant; (ii) the model parameterizations have to reach the same steady state (although they can be characterized by different dynamics), where the steady state fluxes of the system are calculated by using FBA (see Section 3.2).

EM is more appropriate than other approaches (e.g., PE and RE methods (see Section 2.2 and Section 2.3)) to directly account for uncertainties, especially in the case of (i) models with large numbers of unknown parameters that involve a huge search space, and (ii) when some parameters are not completely identifiable with the available experimental data [102]. As a result, EM can help in characterizing a subset of kinetic parameters that provide an equivalent description of time series concentration data. Examples of EM exploited to address uncertainty in the modeling of metabolic and other biological networks can be found in [101,103,104,105].

Another advantage of EM is that it bypasses the need for a detailed characterization of kinetic parameters, and allows to define a set of models that describe relevant behaviors upon enzyme perturbations. Indeed, EM can capture phenotypes that are dependent on changes in the enzyme levels. Once the en- semble of models is produced, additional experimental data obtained in perturbation experiments—such as flux shift in response to enzyme over-expression, or knockouts on the production rate of any molecular species—are acquired and used to iteratively reduce the set of candidate models, resulting in an increasingly predictive sub-set of models. A similar approach was presented in [26], where Metabolic Control Analysis (MCA)—a mathematical framework that describes how fluxes in a given network depend on network parameters, such as enzyme activity or concentrations—was coupled with a Monte Carlo sampling to identify drug targets in biological networks in the case of parameters uncertainty.

A different application of EM can be found in [106], where the authors proposed an optimization- based algorithm for the systematic identification of genetic/enzyme perturbations, to maximally reduce the number of models retained in the ensemble after each round of model screening. The key premise is to design perturbations that maximally scatter the predicted steady state fluxes over the ensemble parameterizations. The effectiveness of this procedure was applied on a metabolic model of central metabolism in *E. coli*, by successively identifying single, double, and triple enzyme perturbations that cause the maximum degree of flux separation between models in the ensemble. In particular, it was found that, in the case of the analyzed metabolic network, knockouts bring about a larger scatter in the flux predictions with respect to two-fold enzyme over-expression.

As an extension of the original EM approach, in [107] a strategy to address structural uncertainties was presented. In this work, models achieving a desired behavior, but characterized by different structures, were generated to investigate different hypotheses about the possible interactions between molecular species involved in a system.

One of the main limits of the EM approach concerns the availability of experimental measurements or the capability of calculating the steady state fluxes, used as reference for the computational analyses. Being the main input of the models in the ensemble, the lack of these values can therefore straiten the applicability of EM.

## 3. From Models to *in Silico* Data

### 3.1. Topological Analysis

The topological analysis of a metabolic network relies on the information about its *structure*, given in terms of nodes and edges, disregarding any other quantitative parameter about the stoichiometry of reactions, the molecular amounts or the kinetic rates. This “network analysis” is usually applied to interaction-based models, which can represent metabolic maps collected in the KEGG [32] database and opportunely converted into a graph (see Figure 2). The focus of this section is mainly devoted to the use of algorithmic methods of graph theory [108] to systematically investigate and evaluate the topological properties of metabolic networks. These properties generally include the study of *degree distributions* (*i.e.*, statistics on the number of edges connecting a node to other nodes), *hubs* (highly connected nodes in the network), *centrality measures* (stating the relative importance of nodes and edges in the network), *motifs* (specific subgraphs occurring with a high frequency in the network), *clusters* (densely connected subgraphs of nodes), *etc.* [109,110,111]. It is however worth mentioning that several other methods have been proposed for the qualitative analysis of interaction-based models; one example is the application of the backtracking process to estimate the minimal nutrition requirements, as proposed in [112].

Topological analysis can be exploited for several purposes. For example, it can serve to easily find errors or omissions in a reconstructed metabolic network, such as the dead-end metabolites and network gaps that will be discussed in Section 4.1). It can identify the main general features of the structural organization of large-scale networks, and understand the underlying processes at the basis of the evolution of the structure itself [113]. Moreover, topological analysis may help to predict some behavioral aspects of the network, such as the existence of autocatalytic sets of molecules [114].

In this context, an interesting work appeared in [27], where a systematic comparative analysis of the metabolic networks of 43 organisms, representing all three domains of life, was presented. The major results of this analysis underlined that metabolic networks across different species are characterized by the same topological scaling properties, namely, they are *scale-free* networks (*i.e.*, networks where the degree distribution of nodes follows a power-law, that is, most of the nodes have few edges connecting them to the other nodes and only a few nodes are hubs). A fundamental consequence of the scale-freeness property is that the network is robust against random perturbations (node or edge removal), but very fragile if hubs are disconnected [115]. Another general property of metabolic networks (which may be observed in generic scale-free networks) is the *small-world * character [116,117], that is, any two nodes (two substrates) in the network can be connected by relatively short paths (the biochemical pathways) along existing edges. Concerning this issue, the diameter of the network (the maximum distance, calculated for all pairs of nodes in the graph, where the distance indicates the shortest path length between two nodes) was, surprisingly, found to be the same in all 43 analyzed organisms, despite the different number of substrates among the investigated species [27]. Other works expanded topological analyses of metabolic networks, investigating further important properties of large-scale biological networks, such as the bow-tie architecture [118] and modularity [119,120]. In particular, the latter issue can help to reduce the model complexity and, as a consequence, to decrease the computational costs.

More recently, in [121] the issue of metabolic graph representation was addressed, stressing the fact that metabolic networks should be preferably modeled as bipartite graphs, that is, networks characterized by two sets of disjoint nodes (nodes representing the metabolites, interconnected by nodes representing reactions). Indeed, the bipartite representation might yield a view of metabolism that is more adherent to reality, allowing the introduction of stoichiometric information, which is a key feature in constraint-based modeling (see Section 3.2). Starting from evidences obtained from bipartite network studies in other fields, in [121] it was suggested that the topological analysis of metabolism should be revisited, in order to confirm hints emerging from literature. For instance, further analysis carried out on the metabolic networks of the 43 organisms studied in [27], successively highlighted the presence of a hierarchical organization characterized by a high intrinsic modularity of the networks [122].

A special kind of bipartite graph is the Petri net (PN), a mathematical formalism extensively exploited for the modeling, simulation and analysis of the structural and behavioral properties of complex systems. According to PNs naming conventions, the graph is characterized by two kinds of nodes: the places (e.g., metabolites), characterized by a state (e.g., number of molecules), and the transitions (e.g., reactions involving metabolites). PNs are directed graphs, that is, edges connecting places to transitions and transitions to places specifically describe the roles of the chemical species as either reactants or products. A PN representation of a metabolic networks can be straightforwardly converted to a reaction-based model to investigate its kinetic properties [123], or be exploited to analyze the biologically meaningful characteristics of the network [124]. For instance, *invariants* allow to identify conservation relations between chemical species or flux distributions at steady state, while the *liveness* property ensures that all reactions of a metabolic pathway can happen at any time. Further information about PN properties and their application to the study of biological systems can be found in [125].

### 3.2. Flux Balance Analysis

Constraint-based modeling relies on the idea that all the expressed phenotypes of a given biological system must satisfy a number of constraints, which can be of physicochemical, spatial or environmental type. Accordingly, by restricting the space of all possible system’s states, it is possible to determine the functional states that a metabolic network can or cannot achieve. The fundamental assumption of constraint-based modeling is that, regardless of the environmental condition, the organism will reach a quasi-steady state that satisfies the given constraints. A plethora of methods have been developed within this framework and a comprehensive list and description can be found in [126,127].

The starting point of constraint-based modeling is the stoichiometric matrix (see Figure 2), which contains the stoichiometric values of the reactants and products of each reaction in the metabolic network: each row in the matrix corresponds to a metabolite, each column corresponds to a reaction. The null space of this matrix mathematically represents the mass balances for each intracellular metabolite, and expresses all those flux distributions that can be achieved by the metabolic network at steady state. Since metabolic networks typically include more reactions (hence fluxes) than metabolites, the stoichiometric constraints alone lead to an under-determined system in which a bounded solution space of all feasible flux distributions can be identified. Additional constraints, such as irreversibility or capacity constraints, should then be incorporated to further restrict the solution space, by specifying the maximum and minimum values of the flux through any given reaction. Capacity constraints are usually set according to experimental records and are recommended at least for nutrient intake reactions.

FBA [128], which is probably the progenitor of all constraint-based computational methods, allows to select a single flux distribution within the obtained feasible solution space, by assuming that the cell behavior is optimal with respect to a specified objective, and by calculating the optimal flux distribution by means of an optimization routine. The objective is represented according to an *objective function* (OF) that, in conventional FBA, takes the linear form z = $\sum_{i\text{=1}}^{n}$*c_i_v_i_*, where *c_i_* is a weight indicating how much the flux *v_i_* of reaction *i* contributes to the OF.

To summarize, the inputs of FBA are the stoichiometric matrix, the specification of the flux boundaries for each reaction and the OF, whereas the output is the quantification of the flux through each reaction. As long as the OF is a linear equation and all constraints are linear (dis)equations, FBA constitutes a linear programming problem; nevertheless, non-linear FBA variants were also formulated (a complete review of the optimization techniques for FBA can be found in [129]). An interesting example of non-linear OF was provided in [130], where it is assumed that *S. cerevisiae* minimizes the number of active reactions necessary to produce the required biomass.

FBA was originally developed within the scope of metabolic engineering where the objective represents some bioengineering design goal, such as maximization of the production of a biochemical compound of industrial use (e.g., biofuels [131]). However, thanks to the advantages of a modeling approach that does not require information about kinetic parameters, FBA has recently received increasing attention in Systems Biology, to gain novel knowledge about the physiological state of a cell. In this context, moving from the postulate that organisms have developed control structures to ensure optimal growth in a constraining environment, the assumption of maximization of biomass yield as the OF has been successful in predicting some phenotypical characteristics of microorganisms [132]. Another commonly used OF is the maximization of ATP production [61,133] to investigate mitochondrial energy metabolism. In this regard, an interesting example devoted to the investigation of the Warburg effect was presented in [61]. This study takes into account an enzyme solvent capacity constraint and it is performed on a CM of cell metabolism. The results showed that, at low glucose uptake rates, mitochondrial respiration is the most efficient pathway for ATP generation; on the contrary, above a threshold of glucose uptake rate, an increase of the glycolytic activity, together with a boost of the flux towards lactate excretion and a gradual decrease of the mitochondrial respiration, allow for the highest rate of ATP production.

Given a certain OF, FBA tries to find a unique optimal solution; however, it is possible that, even for small networks, there exist multiple flux distributions resulting in the same optimal objective value. As different optimal flux distributions could lead to distinct biological interpretations, some strategies were proposed in order to deal with this problem. A first class of approaches tries to identify all flux distributions characterized by the same “best” objective value. To this end, Elementary Flux Modes [134] and Extreme Pathway Analysis [135] are frequently exploited to analyze the entire flux space. Unfortunately, the number of elementary flux modes grows exponentially with the number of reactions in the network, so that their enumeration becomes intractable for genome-scale networks. However, some strategies to make this computation possible have been proposed [136,137]. The second class of approaches makes use of Flux Variability Analysis (FVA), which, instead of listing all the alternative flux distributions, indicates the range of variation of each flux. The method identifies the maximum and minimum values allowed for the fluxes through each reaction, by constraining the objective value to be close or equal to its optimal value [138]. Since the application of FVA to GW models is often limited by a high computation time, tools to improve its efficiency have been developed (see, e.g., [139]).

As a general remark, solutions obtained with FBA are only as good as the constraints used to identify them. Moreover, an appropriate OF is of pivotal importance in FBA studies to determine the physiological state of a cell. The identification of a plausible OF is a delicate task: knowledge about the true OF that drives the evolution is indeed limited, especially when dealing with multicellular organisms. Besides, even when the maximization of biomass yield is assumed, the stoichiometric coefficients of the biomass precursors—as well as the cofactors requirement to synthesize the monomers from the precursors—must be appropriately defined according to the requirements necessary for proliferation, using literature values of experimentally determined biomass composition [140]. Furthermore, even a precise formulation of the OF does not allow to exclude that the organism is in a sub-optimal state. For the above mentioned reasons, new approaches are emerging for an unbiased analysis of the solutions space, aimed at describing global network properties [65,141].

A major limit of FBA is that it is not suited for the investigation of the system dynamics, as it disregards information on metabolic concentrations and kinetic parameters. An attempt to solve the problem is provided by Dynamic Flux Balance Analysis (dFBA) methods, which are intended to partially capture metabolic dynamics by incorporating, according to experimental evidences, the modifications of flux boundaries over time due to the change in the uptake of molecules induced by nutrients availability, or genetic expression effects on transporters. Two methods within this category are: (i) Dynamic Optimization Approach [142], which exploits a non-linear optimization over the whole evaluated time interval in order to derive flux distributions and metabolite levels; (ii) Static Optimization Approach [142], which is performed by dividing the evaluated period into small intervals, optimizing via linear programming at the beginning of every single interval, and then integrating over the entire time interval.

Despite all the aforementioned challenges, FBA has significant potential to support the interpretation of increasingly available, yet non-comprehensive, metabolic data, and there have been many innovative developments to improve its predictive capabilities [129]. In particular, an interesting opportunity is offered by the possibility of integrated analyses of regulatory and metabolic networks [143,144], where regulation of gene expression is described by means of Boolean functions, in which a gene is “on” if the rule is satisfied, “off” otherwise. If a gene is repressed, the fluxes of reactions involving the corresponding protein product will be constrained to zero. A more sophisticated attempt to integrate the gene products in various intracellular signaling, metabolic, and regulatory reactions was presented in [145]. The interested reader can also refer to [146] for a thorough survey on the integration of transcriptomics data into GW constraint-based models of metabolism.

An emerging research front that exploits FBA and that deserves a final remark has the purpose of identifying the topological features of a metabolic network. For instance, groups of reactions in a network that always appear together in functional states were identified by means of flux coupling finder [147] and Extreme Pathway Analysis [148]. Evidence of a robust modular organization at the metabolic level was supplied also for optimal growth in [149] and for optimal nitrogen fixation in [120].

### 3.3. Simulation of the Dynamics

The simulation of a biological phenomenon through a mathematical model allows to obtain the (quantitative) temporal evolution for each molecular species occurring in the model itself (see Figure 2). Simulations can only be realized when all the following information are known: (i) the functional interactions between the species involved in the modeled biological system, (ii) the kinetic parameters associated to the chemical reactions occurring in the system (e.g., reaction rates, turnover numbers, membrane potentials, *etc.*), and (iii) the initial state of the system (*i.e.*, the initial molecular concentrations of the species, charges distribution, *etc.*). As a consequence, the simulation of the dynamics can be generally done for (small-scale) mechanism-based model.

A main advantage of simulation algorithms regards the opportunity of easily testing the behavior of the system under different perturbations. For instance, by increasing (decreasing) the value of a kinetic constant, the system can be simulated in the condition corresponding to an enhanced (reduced) activity of that chemical reaction. Moreover, the initial state of the system can be varied as well, hence mimicking the biological conditions which range from the deletion to an arbitrary *n*-fold over-expression of one or more chemical species. In general, it is possible to automatically perturb each model parameter to systematically analyze the corresponding system response, in order to gain insights into the underlying biological process in a very reduced time with respect to laboratory experiments.

Simulations can be realized by exploiting different computational tools, which can be chosen according to the scale of the modeled system, to the nature of its components and to any possible role played by biological noise. In what follows, we provide a brief description of the most used simulation methods for metabolic processes modeled as systems of differential equations.

**Ordinary differential equations.** When the molecular species involved in a system are present in large amounts, as it is usually assumed in metabolic networks, the effect of noise can be neglected. In these case, it is therefore convenient to apply a deterministic modeling approach, where each molecular species is described by means of a continuous variable (representing its cellular concentration) and its variation in time is formally described through ODEs. ODEs represent the standard mathematical framework for the simulation of the dynamics of biological systems: given the initial state of the system (*i.e.*, the concentration values of all species), together with the kinetic parameters, the dynamics of the system is usually obtained by numerically solving the set of differential equations. Being one of the most applied approaches in almost any area of the science, there exist many efficient numerical integrators to rely on [150,151].

Mechanistic models based on ODEs can give a detailed description of the metabolic pathway under investigation, or even of the functioning of some specific molecular components, thus facilitating the investigation of the relationships between the modeled system and various related diseases. For instance, in [152] the functioning of the mitochondrial adenine nucleotide translocase was analyzed in details by exploiting an ODE-based model and using PE and sensitivity analysis methods (see Section 2.2 and Section 4.2, respectively). In particular, the authors investigated the antiporter behavior under close-to-physiological conditions, predicting some characteristics that are difficult to measure experimentally, such as the dependence of the exchange rate on the membrane potential and on metabolites concentrations. More recently, [153] described the electron flow through the transmembrane protein cytochrome *bc*_1_ complex (also called complex III), leading to the production of reactive oxygen species (ROS) involved in the pathophysiology of several diseases. This small-scale mathematical model was able to reproduce the experimental data of the activity of the mitochondrial electron transfer chain, and allowed to investigate the response of complex III in the presence of the inhibitor antimycin A, as well as the effect of different values of the membrane potential on ROS generation. The behavior of the electron transfer chain was also analyzed with a novel composable kinetic rate equation (called the chemiosmotic rate law) in [154], solving some limits of previous ODEs models of the same mitochondrial process [155,156], therefore possibly facilitating the comparative analysis of the function of these structures in different pathological conditions.

On a larger scale, a comprehensive kinetic model of human hepatic glucose metabolism was presented in [157]. This ODE-based model considers the glycolysis, gluconeogenesis and glycogen metabolism in human hepatocytes, integrated with the hormonal control of these pathways by insulin, glucagon and epinephrine. The simulation of this model, consisting in 49 metabolites and 36 reactions over three different compartments (blood, cytosol and mitochondrion), allowed a molecular level analysis of the hepatic carbohydrate metabolism, including hormonal regulation. The results of the simulations highlighted the importance of short term regulation of metabolism by interconvertible enzymes, being able to adapt hepatic metabolism to hormonal signals and glucose levels, and to switch between anabolic and catabolic modes.

In [158], the authors described a model consisting of 34 ODEs to investigate the properties of intracellular glutathione metabolism, focusing in particular on the effects of oxidative stress and trisomy 21. Simulations of this model, under experimental conditions of the over-expression of genes of chromosome 21 and of increased oxidative stress, helped to explain the metabolic profile of Down syndrome. In addition, in the case of increased oxidative stress and adenosine concentration, the model properly simulated the typical metabolic profile of autism.

A model of glycolysis in the parasitic protozoan *Trypanosoma brucei* was presented in [159]. The authors, instead of investigating the behavior of the system given a fixed parameterization of the model, exploited numerical integration methods to perform different analyses, in order to evaluate the effect of parameter uncertainty. In particular, the authors collected experimental data and confidence intervals of all parameters and defined their probability distributions. Despite these uncertainties, the computational analysis of the model allowed to identify the robustness (e.g., the repartition of the glycolytic flux between the glycerol and pyruvate producing branches) and the fragility of the model (e.g., the processes leading to the accumulation of 3-phosphoglycerate and/or pyruvate).

**Reaction-Diffusion models.** The classic modeling approaches for the description of a biochemical system do not usually take into account the diffusion of molecules. However, if the effect of chemical species localization plays a relevant role on the system dynamics (see, e.g., [160]), then the system can be modeled by means of Partial Differential Equations (PDEs), that define a Reaction-Diffusion (RD) model [161,162,163]. PDEs provide a continuous time and space domain description of the system, where the mass transport, the chemical kinetics and the conservation laws, together with the boundary conditions, are embedded within the same set of equations that can be solved analytically or numerically.

Through the development of a RD model, in [164] the authors analyzed the effects of the autocatalytic release and diffusion of the ROS superoxide on the establishment of membrane potential depolarization waves in mitochondria networks. Model simulations matched the membrane potential depolarization wave propagation, that were experimentally observed in heart cells in response to oxidative stress, and suggested that there exists a minimal number of mitochondria sufficient to trigger cell-wide responses through ROS-dependent coupling of mitochondria in the network. The computational results showed that a few oscillating mitochondria are able to entrain the entire network into an oscillatory regime, even if most mitochondria are not in the oscillatory parametric domain.

In [165], a RD model of aerobic metabolism in skeletal muscle based on PDEs was presented. In particular, this RD model incorporates a detailed description of oxidative phosphorylation in the mitochondria, coupled to the intracellular ATPase reactions, and includes both creatine kinase and myoglobin. The results of the simulations allowed to investigate the role of phosphagen kinase reactions on the diffusion limitations of skeletal muscle fibers, to verify the role that creatine kinase has in buffering ATP concentration (and that myoglobin has no similar effect on free oxygen concentration), and to assess that aerobic metabolism in skeletal muscle is generally not diffusion-controlled.

Different simulation and computational methods can be integrated each other, as proposed in [166,167], where the authors introduced an approach to complement the simulation of dynamic models with the result of static analysis (based on Metabolic Flux Analysis, MFA) of potentially large-scale models of metabolism. This method could facilitate the reduction of the number of biochemical experiments (needed to determine the kinetic parameters) by replacing the information of suitable enzymatic reactions with a MFA-based static module, which only requires the availability of stoichiometric coefficients. This hybrid dynamic/static algorithm showed good results, although inconsistencies might occur when (i) there exist many bottleneck reactions, that is, boundary reactions that cannot be easily assigned to—or simulated by—either the static or the dynamic modules; (ii) bottleneck reactions are not clearly identifiable; (iii) there are large fluctuations in the rate of reactions included in the static module.

The simulation methods described above share a common limitation related to the high level of details of the information required to properly set up the model, posing serious constraints on the size (in terms of metabolites and reactions) of the biological system that can be investigated. However, many efforts were focused on the acceleration of these algorithms, for instance by using special hardware like field-programmable gate arrays [168], computers clusters [169] or GPUs [170,171]. This holds also for stochastic algorithms and hybrid deterministic/stochastic simulation methods, which are usually not applied for the study of metabolism and are therefore not discussed in this work. Nonetheless, it is worth mentioning that recent experimental results evidenced that stochastic fluctuations in the expression level of metabolic enzymes could actually cause fluctuations in cell growth [172].

## 4. From *In Silico* Data to Experimental Hypothesis

In this section we present different computational strategies to perform thorough analyses of metabolic models. The outcome of these methodologies can be exploited to gain new insights about the functioning of a metabolic pathway, and to formulate novel hypotheses about its behavior in different conditions. This usually leads to the design of *ad hoc* model-driven experiments, along with the possible definition of new laboratory protocols, in order to validate the computational hypotheses.

### 4.1. Model Validation

Any *in silico* metabolic model must undergo a validation process (Figure 2) to confirm its capability to reproduce the behavior and properties of the biological system under study in different conditions.

**Figure 4:**
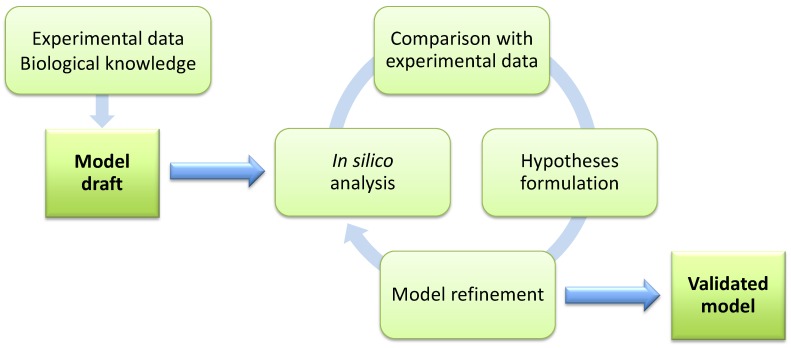
Schematic representation of the validation process of mathematical models. A model draft undergoes an iterative process in which the *in silico* outcomes are compared to the experimental data to validate the model, and to formulate new hypotheses about the functioning of the underlying biological process. A validated model can then be used for deeper computational investigations.

The evaluation of a model proceeds through the analysis of its behavior (as described in Section 3), which allows to detect problems in its reconstruction, according to an iterative process of tuning, simulation and validation (Figure 4). Errors in a metabolic reconstruction are more likely to occur within large-scale models, which are typically investigated with constraint-based approaches. In this context, some known issues that can affect the validity of a metabolic reconstruction are the presence of dead-end metabolites, *i.e.*, metabolites that are only produced or consumed within the network, or the existence of network gaps [29], which are missing reactions that should connect some metabolites. Another major issue concerns the detection and correction of thermodynamically infeasible loops [55,56]. In addition, the validity of mechanism-based (dynamic) models is typically challenged by the uncertainty of the values of the model parameters, an aspect that will be widely discussed in Section 4.2.

Metabolic models might be evaluated: (i) qualitatively, e.g., to assess their capability to generate all the precursor metabolites, with particular regard to biomass components, and/or all the metabolites that the organism is known to produce or degrade; (ii) quantitatively, by comparing the model behavior with various experimental observation such as secretion products and gene essentiality. For example, in [173] the results of a FBA on a genome-wide reconstruction of *A. niger* were validated against experimental data in literature regarding yields of different products and biomass on substrates. Transcription data were also utilized to assess the network topology and the validity of gene annotation. The analysis of the physiological responses of a model, such as growth as a function of glucose supply [173,174], is also widely used.

The strategy of *gene deletion analysis* deserves particular attention as it might be exploited either to validate a model, or to infer novel experimental hypotheses after model validation. This method consists in simulating the inhibition of a metabolic gene by excluding from the model the reactions associated to that gene. For example, in [175], FBA was applied to calculate the maximal biomass yields on glucose for all possible single gene deletions in the central metabolic pathways of *E. coli*. The model was able to qualitatively predict the growth potential of mutant strains in 86% of the examined cases. Along similar lines, the effect on cell viability of 87.8% of 599 single gene deletion experiments in a genome scale model of *S. cerevisiae* resulted in agreement with experimental observations when growth on synthetic complete medium was simulated [176].

### 4.2. Sensitivity Analysis

The behavior of mathematical models of biological systems, especially in the case of complex metabolic pathways, highly depends on the values of the parameters (e.g., kinetic constants of the reactions, molecular concentrations, flux boundaries) that characterize them. Model outcomes may therefore present a substantial variability due to (i) a large uncertainty, associated to some input parameters, that can propagate throughout the model; (ii) a possible intrinsic sensitivity of the model, such that small changes in the input result in large changes in the output.

The source of uncertainty in the definition of the model inputs can derive from errors in the experimental measurements or, more often, from lacking or contradicting information present in literature. A clear example is given in [158], where the Michaelis-Menten constants were taken from literature to develop a mathematical model of one-carbon and glutathione metabolism. The supplementary materials of this work show the range of values obtained for each parameter: for example, the *V_max_* of 10-formyltetrahydrofolate synthase varies between 100 and 486,000 *µ*M/h.

In the situations described above, a computational analysis aimed at characterizing the relationships between model input and model output might be fundamental: sensitivity analysis (SA) represents a methodology well suited for this purpose (Figure 2). Given the topology of the biological network under investigation, a reference parameterization, and a range of variability for the parameters involved, SA allows to quantify how much a given property of the system depends on the variation or on the uncertainty in its parameters [177,178]. It is worth noting that SA markedly differs from simple *parameter sweep analysis*, whose outcome is a qualitative visualization of the relationship between model input and output. In fact, the outcome of SA are *sensitivity indexes* (or coefficients) that represent the “importance” of each parameter and allow to rank them, with higher values indicating those parameters the model is most sensitive to. Remarkably, a ranking of the parameters can bring about useful insights at different stages of the modeling process and for several applications [178].

At the stage of model calibration, SA can provide indications on which parameters require a precise knowledge in order to reduce the uncertainty in the model output. On the contrary, the least sensitive parameters could be disregarded from the model: indeed, SA can support model reduction, an important step to eliminate redundancy in the models and to uncover the most important control mechanisms of biological systems. For instance, in [179], the identification of parameters ranking according to SA results, allowed to reduce a dynamic model of the glycolysis and the pentose phosphate pathway of *E. coli* from 122 to 22 reactions without significant discrepancies in the model predictions.

SA allows to investigate the robustness of the model and of its predictions, but perhaps its most important application is related to MCA [180], a theory devoted to the analysis of the distribution of control within a network. This theory can be applied also in the context of the network-based drug design, where MCA coupled with Monte Carlo sampling [26] was employed to identify the fragility points of a network, to asses their influence over different physiological states, and to maximize the drug response while avoiding toxicity.

Sensitive parameters identified by means of SA may indeed represent either control points of the system behavior, or spots susceptible to failure. Identification of these parameters can therefore guide the design or facilitate the choice of which are the most suitable control experiments to carry out, thereby reducing laboratory costs and efforts. For example, in [181] a SA of a dynamic model of metabolism in glucose- and palmitate-perfused rat hearts revealed that control of metabolism is distributed among a number of enzymes. It was also shown that palmitate utilization is mainly controlled by citrate synthetase, succinate dehydrogenase and malate dehydrogenase, in contrast with the simplistic hypothesis derived from experimental observations that palmitate utilization was limited by the rate of acetyl-CoA oxidation in the TCA cycle.

Among the existing SA techniques [182], in the context of metabolism *local SA* methods have been mainly applied [179,181,183,184]. Local SA methods investigate the behavior of the model in the immediate region around a reference value (referred to as *baseline * value) of the model parameters. Typically, they exploit *partial derivative coefficients*, which can be estimated either numerically, by finite difference approximation (see, e.g., [183,184]), or analytically, by developing a second set of differential equations to be solved, which describe the sensitivity of the original system with respect to the chosen parameter (as, e.g., in [181]).

Local SA is appropriate when the parameter values are measured with *ad hoc* experiments, and the uncertainty is thus related to the experimental error, or when the model is already validated, and there is a good confidence about the parameter values. However, parameter uncertainty is often uncontrolled, especially when parameters values are taken from literature, or when they are inferred via PE methods (see Section 2.2). In such situations, *global SA* methods, which investigate the behavior of the model within the entire variation range, should be preferred. Global SA usually exploits variance-based methods (a review can be found in [185]), which assess the sensitivity indexes in terms of the fraction of variance in the output that can be apportioned to the variance in a given input. Examples of global SA of metabolic models are nevertheless rare in literature [186], due to the prohibitive computational costs it requires. As a matter of fact, SA, like parameter sweep analysis, usually requires the execution of one (or more) simulation for each parameterization of the model under investigation. In this regard, local methods allow to vary one input parameter at a time (OAT), while keeping all others at their baseline values. On the contrary, global SA methods fully explore the input space, also taking into account mutual interactions between parameters. In this case, the number of possible parameterizations grows exponentially with the number of parameters, and sophisticated statistical techniques are necessary to efficiently sample the input space (e.g., the latin hypercube sampling strategy [187]). It is worth noting that there exist global SA methods that take advantage of the simplification OAT, by calculating local measures in different points of the parameters space (e.g., the elementary effect method [182], or the procedure proposed in [188]).

Although the computation costs associated to the number of possible parameterizations can become very high, it is worth noting that the simulations needed for the computation of the sensitivity indexes are independent from one another and can thus be performed in a parallel fashion. Therefore, parallel architectures such as computer clusters or GPUs can be exploited to drastically reduce the overall running time.

### 4.3. Control Theory

Recently, several theories originally developed within scientific fields of economy or engineering—such as game theory, control theory, systems theory, circuits analysis, signal processing, *etc.*—have been applied for the investigation of biological systems (see, e.g., [189,190,191,192,193,194,195,196]). Thanks to their potentiality, these theories can facilitate the comprehension of the architecture of cellular processes, and shed light on the different layers of regulation that exist at all levels of cell functioning [197,198].

In particular, the goal of control theory is to identify the nature of the control structures that regulate the behavior of cellular systems, such as feedback, feedforward and cascades [199] (Figure 2). Feedback control is both a powerful and a potentially dangerous strategy to regulate either the internal variations or the environmental interferences on the cell. For instance, negative feedback is able to promote stability and robustness by ensuring that variations in temperature, concentrations, binding affinities, *etc.*, do not hamper or impair the cell functioning. On the contrary, positive feedback (feedforward) deliberately destabilizes the system equilibrium by increasing the difference between the current and the previous system’s response, as in (second messenger-mediated) signal amplification in transduction pathways.

In this context, some recent works highlighted the feasibility of control theory for the analysis of different aspects of metabolism. These works usually rely on small-scale mechanistic models of metabolic pathways, described by means of ODEs. In [200], for instance, a detailed analysis of feedback, feedforward and cascade control structures was carried out in metabolic networks to clarify the basic features of glycolysis and oxidative phosphorylation, and their response to various changes in energy demand. By assuming different hypotheses for the regulation of energy metabolism, the authors reproduced the common emerging properties of energy metabolism, such as oscillations and homeostasis. This type of analysis is of peculiar importance as it might allow to comprehend, at a molecular scale, the exact mechanisms of regulation that are involved in metabolic processes, and could therefore facilitate the comprehension of pathological conditions [201]. A novel perspective on feedback regulation in cellular systems was recently presented in [202], where a coupled feedback-feedforward scheme was analyzed to explain the way two global transcription factors, ArcA and Fnr, are able to coordinately regulate anabolism, catabolism and chemiosmosis in *E. coli*. Interestingly, the analysis was based on ChIP-chip and gene expression data, interfaced with a curated GW metabolic model of *E. coli*. Thanks to this approach, the authors showed that the discovered feedback-feedforward architecture between ArcA and Fnr is able to control the metabolism of *E. coli* at the genome level.

It is now generally accepted that many problems in Systems Biology have analogies with problems in control theory. Though, biological systems are much different from engineering systems and require an accurate application of standard analysis methods. For instance, cellular processes are far from being linear systems, they are not continually at steady state conditions, and there is usually a lack of adequate or sufficient information about their kinetic parameterization. Therefore, classical methods of control theory still need to be challenged by novel questions in order to be fully applicable and predictive for biological systems [196,198,203].

## 5. Computational Strategies at Work: Gaining Novel Insights on Metabolism

The methods and procedures described above offer to computational and systems biologists a vast array of tools to investigate metabolism. Consistently, different questions may be asked. In this section we outline some relevant questions that different metabolic models may help to answer, in both basic and applied research.

### 5.1. Increase, Integrate and Validate Biological Knowledge

For a biologist, the first aim of model building is to rationalize and give structure to existing knowledge that may be scattered through a large number of papers and databases, which are difficult to integrate and often contain disparate and/or contradictory data sets. A typical example of this type of application is the construction of GW models, that allow to refine metabolic maps, to resolve contradictory data, to fill empty spaces in the reconstructed networks and iteratively build more and more precise maps.

An outstanding case of a model increasing and validating biological knowledge can be found in [204]. In this work, starting from a database of 900 different sources, the authors proposed a whole-cell model of *M. genitalium* integrating 28 submodels of cellular processes, ranging from metabolism and transport to gene expression, DNA replication and cell cycle control. Each of these processes was simulated independently on a short time scale using an adequate computational strategy (e.g., metabolism was analyzed by means of FBA), and subsequently integrating the results with those obtained on longer time scales with a numerical integration algorithm.

On the whole, this model was efficiently employed to improve the biological knowledge on *M. genitalium*. In particular, in the domain of metabolism, the model correctly predicted that flux through glycolysis is more than hundred times higher than the flux through pentose phosphates and lipid biosynthesis. Moreover, the authors identified the availability of dNTPs as a cell cycle regulator, highlighting a strong connection between cell cycle and metabolism. Finally, from the energetic point of view, the model suggested that the production of mRNA and proteins are the most consuming ATP and GTP processes, and it also evidenced a relevant discrepancy in energy production and consumption due to cellular maintenance costs and futile cycles.

### 5.2. Generate Experimentally Testable Hypotheses: Identify Regulatory Nodes and Drug Targets

The comprehension of the complex regulatory properties of metabolic networks would be of great benefit in many fields, including the ability to properly fine tune and/or control fermentation of industrially relevant yeasts, or the clarification of metabolic alterations in multifactorial diseases, such as cancer. From a metabolic point of view, the budding yeast *S. cerevisiae* and tumor cells share several features, since both yeast and cancer cells preferentially use fermentation rather than respiration to generate ATP. These metabolic behaviors—referred to as Crabtree and Warburg effect in yeast and cancer cell, respectively [66,205]—are seemingly counterintuitive, given that aerobic glycolysis yields much less ATP molecules per moles of glucose than mitochondrial respiration. Vasquez *et al*. developed a toy model of ATP production that includes stoichiometric reactions for glycolysis, oxidative phosphorylation and biosynthetic reactions [61]. The results obtained with FBA, constrained by the glucose uptake and cytoplasmic solvent capacity, suggest that mitochondrial respiration is the most efficient ATP generating pathway only at low glucose uptake rates. On the contrary, when a threshold uptake rate is exceeded, a concurrent gradual activation of aerobic glycolysis and slightly decreased mitochondrial respiration originate the highest rate of ATP production.

Molenar *et al*. developed a different toy model that integrates several subsystems within a complete self-replicating system [60]. They show that the shift in metabolic efficiency originates from a trade-off between investments in enzyme synthesis and metabolic yields for alternative catabolic pathways. The importance of energy cost involved in synthesis of mitochondria for the respiratory/fermentation switch was also reported in a different FBA study [130]. These results offer new perspectives in the study of metabolism evolution and may have direct relevance for the development of novel chemotherapeutic strategies targeting cancer metabolism.

Despite extensive experimental investigation, the molecular bases of the Warburg effect still remain elusive. Resendis-Antonio and coworkers designed a CM, encompassing glycolysis, TCA cycle, pentose phosphate, glutaminolysis and oxidative phosphorylation, and analyzed its behavior using FBA [67]. Based on these computational studies and their comparison with kinetic analysis of human cancer cells, the authors concluded that specific enzymes (such as lactate dehydrogenase and pyruvate dehydrogenase) play a pivotal role in cancer cell growth. Shlomi *et al*. [206] used the GW human reconstruction Recon 1. In this work, the maximization of the flux through a pseudo-reaction of biomass production allowed the authors to show that, for high fluxes of glucose uptake, aerobic glycolysis is the optimal strategy. It is worth stressing that the Warburg effect would not be observed with standard FBA (*i.e.*, strictly stoichiometric analysis), but a constraint on enzyme solvent capacity was required. The authors reported that their analysis match the metabolic behavior observed experimentally during oncogenic progression of ras-transformed cells. Extension of this work allowed to detect the Warburg effect using GW metabolic models of each of the NCI-60 cell lines and to identify its association with cell migration [207]. These results indicate that both GW models and CMs can be used to study major regulatory features of metabolism, and that different declinations of FBA are extremely valuable in this regard.

By coupling transcriptomic data to Recon 1, Folger *et al.* constructed a generic GW cancer model, which was validated by correctly identifying genes essential for cellular proliferation in cancer cell lines [208]. The availability of models specifically developed to describe healthy and cancer cells, allowed the use of FBA to recognize genes whose deletion would differentially affect growth of the “cancer” model, but not that of its “healthy” version. In addition, the authors showed that a large proportion of the products of the identified genes were targets for approved or experimental cancer drugs. A further work considered the experimental evidences that a knockout in the fumarate hydratase-encoding gene is responsible for hereditary leiomyomatosis and renal cell cancer. Two Recon 1 cell line-specific models constrained by transcriptional data allowed to predict that the haem oxygenase 1-encoding gene is synthetically lethal with fumarate hydratase deficiency, as later confirmed experimentally [209].

Nam *et al*. predicted putative oncometabolites that could result from the loss-of-function or gain-of-function mutations of metabolic enzymes in cancer cells by constraining Recon 2 GW models with large-scale mutational and gene expression data, indicating that constraint-based GW models have the potential for discovering novel oncometabolites in different cancers [210]. Constraint-base modeling was also successfully applied to guide strategies for increasing antibiotic efficiency [211], as well as to devise novel approaches to antibiotic discovery [212].

As in the case of GW models, also CMs can be effectively exploited in the identification of potential drugs to target cancer metabolism. In [213], the study of the dynamics of a kinetic CM of glycolysis provided system-level insights into the dynamic regulation of glycolysis by unraveling the role of full trehalose cycle in yeast (and of futile cycles in general) in balancing upper and lower glycolysis and hence in supporting growth. Model predictions have effectively addressed the experimental manipulation of the size of sub-populations of non-growing cells. In the work presented in [214], the authors exploited EM to define a kinetic model of cancer metabolism in human colorectal cells. EM was used to gain insights and predict potential drug targets for tumor cells. To be more precise, the model was perturbed by under-expressing enzymes in the network, and those showing the greatest decrease in biomass production were considered as potential drug targets.

### 5.3. Design Microbial Strains for Metabolic Engineering and Industrial Applications

Metabolic engineering is the directed genetic modification of cellular reactions. It aims to alter the metabolic architecture of microorganisms to efficiently produce target chemicals [215]. The successful implementation of industrial scale metabolic engineering could greatly benefit from *in silico* strategies able to guide strain development [216]. GW metabolic models were shown to be useful for initial guiding of metabolic engineering [131,217,218], and to construct a pipeline that resulted in the construction of a non-intuitive *S. cerevisiae * cell factory over-producing succinic acid [57,58]. Another application was presented in [219], where an *E. coli* strain that produces 1,4-butanediol (BDO) at high yields was designed. BDO is an important commodity chemical, that is massively produced to satisfy global markets but that is not occurring naturally in any known organism. Putative biochemical pathways able to convert an endogenous *E. coli* metabolite to BDO were identified, and the final pathway was chosen on thermodynamic grounds. A GW metabolic model guided *E. coli* engineering, so that it could produce enough reducing power to drive the BDO pathway starting from either pure sugars or biomass-derived mixed sugars. These works show that *flux optimization* (via FBA) of GW models plays an important role in strain design and development, allows to identify novel and non-intuitive metabolic engineering strategies, and enables new bioprocesses even for commodity chemicals not naturally produced by living cells.

In the context of metabolic engineering, an ODE-based dynamic model describing the metabolic pathways involved in acetone-butanol-ethanol production by *Clostridium saccharoperbutylacetonicum* N1-4 was proposed in [184]. The CM of this metabolic pathway considered two distinct characteristic phases (*i.e.*, acidogenesis and solventogenesis), and allowed to achieve a correct dynamics of metabolites even under perturbed initial conditions, proving the validity of the model for further analyses and its potentiality to formulate predictions about the existence of metabolic bottlenecks for high production of butanol. Similarly, in [183] the authors proposed an ODE-based dynamic model of xylose metabolism in *Lactococcus lactis* IO-1, in which the competitive/uncompetitive inhibition by lactate and the substrate activation/inhibition by xylose was integrated in the rate equations. In both cases, the CMs allowed to correctly simulate the metabolites dynamics, and were used to gain biological insights by means of additional analysis based on sensitivity methods.

Finally, also SA methods can be applied within the field of metabolic engineering. For instance, in [183,184], the SA of a kinetic model allowed to identify the most important pathways for high production of a target product, namely, lactic acid fermentation from xylose and butanol production, respectively.

## 6. Conclusions and Perspectives

In the last decades, mathematical modeling and computational methods have become an indispensable mean to achieve a thorough understanding of the functioning and for the identification of the design principles of complex systems. In general, the mechanism-based modeling approach is characterized by the greatest predictive capability concerning the functioning of biological systems at the molecular level. Anyway, in the context of metabolism the applicability of this approach is limited because of its unsuitability for the analysis of systems with a large size, such as metabolic networks, which are characterized by a high number of metabolites and reactions. On the contrary, interaction-based models and, especially, constraint-based models are the most widely used for the study of metabolism, although they neglect most of the quantitative and kinetic information. To provide a bird’s eye view of mathematical models and the respective computational methods applied for the study of metabolism, we provide in Table 1 a selection of works that appeared in literature in the recent years, some of which have been discussed in this review. In addition, a list of the main software tools used for the investigation of metabolism is given in Table 2.

Irrespective of the chosen modeling approach and of the scale of the model, different computational methods are usually exploited together for the analysis of the system. For instance, reverse engineering techniques usually require parameter estimation, which both rely on algorithms for the simulation of the dynamics of the system [93].

Unfortunately, given a specific computational task and the available methods to solve it, it is not feasible to provide a realistic comparison of the various techniques by defining some *ad hoc* comprehensive quantitative measure. As a matter of fact, the performance of each methodology is strongly affected by the size and the complexity of the model under investigation, as well as by the quality of the available experimental data. Therefore, general quantitative discussions concerning the goodness of a method with respect to similar methods cannot be provided, unless all these methods are applied to the study of the same model.

**Table 1:** Overview of some recent literature papers on the modeling and computational analysis of metabolism.

Pathway/Aim ofthe Model	Cell Type/Organ	Organism	Modeling Approach &Methodology	ExperimentalData	Reference
Glycolysis	-	*T. brucei*	CM, ODE	L	Achcar *et al*. [159]
GW metabolic network and succinic acid production	-	*S. cerevisiae*	GW, FBA	M	Agren *et al*. [58]
GW metabolic network	-	*A. niger*	GW, FBA	L	Andersen *et al*. [173]
Mitochondrial energy metabolism, Na^+^/Ca^2+^ cycle, K^+^ cycle	Heart, liver	*B. taurus, S. scrofa, R. norvegicus*	CM, DAE, PE, SA	L, M	Bazil *et al*. [80]
OXPHOS	Cardiomyocytes	*R. norvegicus*	CM, ODE	L	Beard [156]
Electron transport chain	Heart homogenates	*R. norvegicus*	CM, ODE, CRL	L, M	Chang *et al*. [154]
Glycolysis, OXPHOS	Not specified	Eukaryotic, *H. sapiens*	CM, Control theory	L	Cloutier *et al*. [200]
Bow-tie architecture of metabolism	Not specified	*H. sapiens*	GW, Topological analysis	L	Csete *et al*. [118]
Central metabolism	-	Yeast	CM, FBA	L	Damiani *et al*. [65]
Energy metabolism	Skeletal muscle cell	Mammal	CM, PDE	L	Dasika *et al*. [165]
Glycolysis and pentose phosphate pathway	-	*E. coli*	CM, ODE, SA	L	Degenring *et al*. [179]
Glycolysis and pentose phosphate pathway	-	*E. coli*	CM, ODE, SA	L	Degenring *et al*. [179]
Biosynthesis of valine and leucine	-	*C. glutaminicum*	CM, ODE, SDE	M	Dräger *et al*. [76]
Anabolic, catabolic, chemiosmosis pathways	-	*E. coli*	GW, Control theory	M	Federowicw *et al*. [202]
Small world behavior of metabolism	Not specified	*H. sapiens*	GW, Topological analysis	L	Fell *et al*. [116]
GW metabolic network	Not specified	*H. sapiens*	GW, FBA	L	Duarte *et al*. [39]
GW metabolic network	-	*E. coli* MG1655	GW, FBA	M	Edwards and Palsson [175]
GW metabolic network	-	*H. influenzae*	GW, FBA	L	Edwards *et al*. [36]
Cancer metabolic networks	Various (NCI-60 collection)	*H. sapiens*	Network reconstruction, FBA, gene (pair) analysis	L	Folger *et al*. [208]
GW metabolic network HepatoNet1	Hepatocytes	*H. sapiens*	GW. Network reconstruction	L	Gille *et al*. [220]
Cytochrome bc1 complex, ROS production	Muscle, heart, liver, kidney, brain	*R. norvegicus*	CM, ODE	L	Guillaud *et al*. [153]
GW metabolic network EHMN	Not specified	*H. sapiens*	GW, Network reconstruction	L	Hao *et al*. [221]
GW metabolic network	-	*S. cerevisiae* S288c	GW, Network reconstruction, FBA	L	Heavner *et al*. [3]
GW metabolic network	-	*S. cerevisiae*	Network reconstruction	L	Herrgård *et al*. [43]
Topological properties of metabolism	-	43 different organisms	GW, Topological analysis	L	Jeong *et al*. [27]
Glycolysis, OXPHOS	-	Not specified	CM, ODE, Game theory	-	Kareva [189]
Whole-cell life cycle model	-	*M. genitalium*	GW, FBA, ODE	L, M	Karr *et al*. [204]
Glycolysis, pentose phosphate pathway	-	*T. brucei*	CM, ODE	L	Kerkhoven *et al*. [64]
Energy metabolism	Colorectal cells	*H. sapiens*	CM, FBA, EM	M	Khazaei *et al*. [214]
GW metabolic network	-	*Synechocystis sp.* PCC 6803	GW, FBA	L	Knoop *et al*. [37]
Glycolysis, gluconeogenesys, glycogen metabolism	Hepatocytes	*H. sapiens*	CM, ODE	L	König *et al*. [157]
Adenine nucleotide translocase	Heart mitochondria	*B. taurus*	CM, ODE, PE, SA	L	Metelkin *et al*. [152]
GW metabolic network	-	*Z. mays* L. subsp. *mays*	GW, Network reconstruction	L	Monaco *et al*. [40]
Xylose metabolism	-	*L. lactis IO-1*	CM, ODE, SA	M	Oshiro *et al*. [183]
GW metabolic network	-	*S. cerevisiae*	GW, Network reconstruction, FBA	L	Österlund *et al*. [222]
GW metabolic network and succinic acid production	-	*S. cerevisiae*	GW, FBA	M	Otero *et al*. [57]
Topological properties of metabolism	-	43 different organisms, *E. coli*	GW, Topological analysis	L	Ravasz *et al*. [122]
One-carbon metabolism, trans-sulfuration pathway, synthesis of glutathione	Hepatocyte	*H. sapiens*	CM, ODE	L	Reed *et al*. [158]
Glycolysis, TCA cycle, pentose phosphate pathway, glutaminolysis, OXPHOS	HeLa cell	*H. sapiens*	CM, FBA	M	Resendis-Antonio *et al*. [67]
Modularity of metabolism	Not specified	*H. sapiens*	GW, Topological analysis	L	Resendis-Antonio *et al*. [120]
GW metabolic network	Not specified	*H. sapiens*	GW, Network reconstruction	L	Sahoo *et al*. [223]
Acetone, butanol and ethanol production	-	*C. acetobutylicum*	CM, ODE, SA	M	Shinto *et al*. [184]
Cancer metabolic networks	Various (NCI-60 collection)	*H. sapiens*	FBA	L	Shlomi *et al*. [206]
GW metabolic network	-	*S. cerevisiae*	GW, FBA	L	Simeonidis *et al*. [130]
Glycolysis	-	*S. cerevisiae*	CM, ODE	M	Teusink *et al*. [62]
GW metabolic network	Not specified	*H. sapiens*	GW, FBA	L	Thiele *et al*. [4]
Primary metabolism	-	*E. coli*	CM, ODE, EM	-	Tran *et al*. [101]
Fueling reaction network	-	*E. coli W3110*	CM, FBA	M	Varma *et al*. [174]
Reduced model of cell metabolism	-	-	CM, FBA	L	Vazquez *et al*. [61]
Small-world property of metabolism	-	*E. coli*	GW. Topological analysis	L	Wagner *et al*. [117]
GW metabolic network	-	*C. glabrata*	GW, FBA	L	Xu *et al*. [38]
Erythrocyte metabolism	Red blood cell	*H. sapiens*	Hybrid: ODE + MFA	-	Yugi *et al*. [166]
Mitochondrial energy metabolism	Various tissues	Mammal	CM, ODE	-	Yugi [224]
Modularity of metabolism	Not specified	*H. sapiens*	GW, Topological analysis	L	Zhao *et al*. [119]
ROS-induced ROS release in mitochondria network	Cardiomyocytes	*C. porcellus*	CM, ODE, PDE, RD, Finite Difference Method	M	Zhou *et al*. [164]

*Abbreviations*. CM: Core model; CRL: Chemiosmotic Rate Law; DAE: Differential Algebraic Equations; EM: Ensemble modeling; FBA: Flux Balance Analysis; GW: Genome-wide model; L: experimental data obtained from literature; M: experimental data measured with *ad hoc* experiments; MFA: Metabolic Flux Analysis; ODE: Ordinary Differential Equations; PDE: Partial Differential Equations; PE: Parameter Estimation; SA: Sensitivity Analysis; SDE: Stochastic Differential Equations.

**Table 2:** Main computational tools used in the modeling, simulation and analysis of metabolism.

Tool name	Purpose	Interaction-based	Constraint-Based	Mechanism-Based	Reference
BioMet Toolbox	Genome-wide metabolic model validation, FBA, probabilistic FBA, gene set analysis		√		[225]
Cobra Toolbox	FBA, FVA, dFBA, gap filling, network visualization		√		[226]
COPASI	Determinstic, stochastic and hybrid simulation, PE, SA, MCA			√	[227,228]
cupSODA	Deterministic simulations on GPUs			√	[171]
Cytoscape	Complex networks visualization and topological analysis	√			[229,230]
FAME	Web based FBA and FVA		√		[231]
FASIMU	FBA, FVA, gene deletion analysis, gap filling		√		[232]
OptFlux	FBA, FVA, EFM, gene deletion analysis		√		[233]
Pathway Tools	GW reconstruction, FBA, gap filling		√		[234]
Raven Toolbox	GW reconstructions, FBA, network analysis and visualization	√	√		[235]
SurreyFBA	FBA, FVA, EFM		√		[236]

**Table 3:** Principal databases collecting biological data or metabolic models, fundamental resources for the investigation of metabolism.

Database	Contents	Reference
BiGG	Genome-scale metabolic networks	[237]
BioCyc	Collection of more than 3000 pathways / genome databases	[238]
BioModels	SBML models of biological processes	[239]
Brenda	Molecular and biochemical information on enzymes	[240]
CellML	XML-based models of biological processes	[241]
Ensembl	Genome browser for genomic information	[242]
ExPASy	Portal to existing databases and tools categorized by life science areas	[243]
GeneCards	Omics data on human genes	[244]
HumanCyc	Human metabolism pathways	[245]
Human Metabolic Atlas	Human metabolism models	[52]
Human Protein Atlas	Human protein expression profiles with spatial localization in tissues and cells	[53]
JWS	Curated models of biochemical pathways and simulation tools	[246]
KEGG	Manually curated pathway maps integrating molecular-level information	[32]

In order for a model to be useful to experimental biologists, it has to strongly interconnect with biological data and *ad hoc* experimental measurements during the computational phases of model construction and validation, as well as to test its predictive capability. In a Systems Biology workflow, the interplay between experimental biologists and modelers is indeed indispensable to understand each other’s requirements and to take advantage of the respective expertise. As an example, experimental biologists would be required to design appropriate laboratory experiments, while modelers should be prepared to develop appropriate modeling strategies and simulation tools. This interplay is then expected to increase the efficacy and broaden the scope of mathematical models in the study of metabolism, and increase our knowledge of metabolism and its regulatory properties.

All in all, it is an exciting moment for metabolism modeling. The availability of vastly improved platforms allowing shorter and shorter computation times on the one hand, and increasingly accurate, sensitive and quantitative metabolomics techniques on the other hand, will allow the development of complex, mechanism-based metabolic models and their interconnection with models of other cellular functions, paving the way to the development of whole cell models [204].

## Figures and Tables

**Figure A1:**
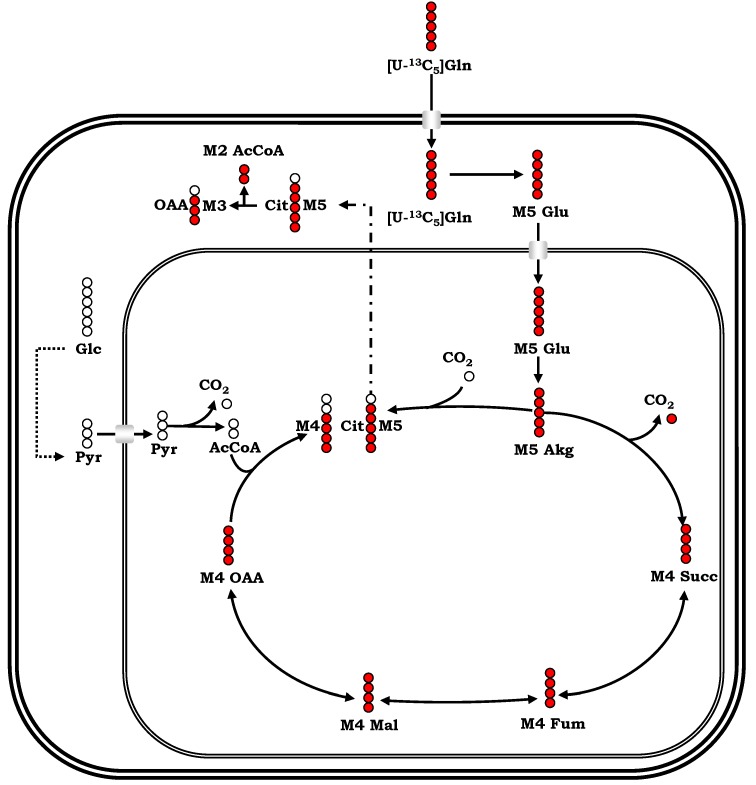
The map illustrates the fate of [U–^13^C_5_]glutamine (Gln) in the TCA cycle. Red (white, respectively) circles denote labeled (non labeled, respectively) carbon atoms in each metabolite. See text for details.

## List of Acronyms

BDO: 1,4-butanediol
CM: Core Model
DE: Differential Evolution
dFBA: Dynamic Flux Balance Analysis
dNTP: Deoxyribonucleotide Triphosphate
EFM: Elementary Flux Modes
EM: Ensemble Modeling
FBA: Flux Balance Analysis
FDG-PET: 18F-fluorodeoxyglucose–positron emission tomography
FVA: Flux Variability Analysis
GA: Genetic Algorithm
GC: Gas Chromatography
GC-MS: Gas Chromatography Mass Spectrometry
GP: Genetic Programming
GPU: Graphical Processing Unit
GW: Genome-Wide
MCA: Metabolic Control Analysis
MFA: Metabolic Flux Analysis
MID: Mass Isoptomers Distribution
MS: Mass Spectrometry
OAT: One-factor-at-A-Time
ODE: Ordinary Differential Equation
OF: Objective Function
PE: Parameter Estimation
PSO: Particle Swarm Optimization
RD: Reaction-Diffusion
RE: Reverse Engineering
ROS: Reactive Oxygen Species
SA: Sensitivity Analysis
SiAn: Simulated Annealing
SDE: Stochastic Differential Equation
TCA: Tricarboxylic Acid

## Appendix I: Experimental Methodologies for Metabolic Data Generation

The metabolome is often identified and quantified using a combination of Mass Spectrometry (MS) with Gas Chromatography (GC-MS) or Liquid Chromatography. In particular, the most popular technology in metabolic physiology and biotechnology is GC-MS, since it requires low amounts of samples to be analyzed, it allows excellent separation and ensures high precision of data [247,248,249]. Several studies have shown that MS analysis, although more expensive, has greater sensitivity for metabolites detection when compared to Nuclear Magnetic Resonance spectroscopy [8,10,250,251,252]. In addition, the recent introduction of the combination of GC and high-resolution mass spectrometry (time-of-flight) with stable isotope tracer labeling, that permits to measure molecular masses in a variety of matrices (gas, liquid, solid) after their conversion into ions, permits unambiguous metabolite assignment. Moreover, the stable isotope tracers (^13^C, ^2^H, ^15^N, ^18^O and ^34^S) are non-radioactive and present no risk to humans, in contrast to radionuclides commonly used in cancer diagnosis, as ^18^F-fluorodeoxyglucose–positron emission tomography (FDG-PET). FDG-PET is clinically used to visualize the glucose uptake of cancer cells, but it suffers of some limitations: it allows to investigate only the first step of glycolysis metabolism, it is able to observe only cancer types with glycolytic phenotype, and it is not able to identify information related to downstream glucose metabolic pathways [253,254]. On the contrary, using appropriate stable isotope tracers we have an effective tool able to characterize the metabolic phenotype of organisms [255,256].

Briefly, the introduction of a stable isotope tracer (such as ^13^C) in the culture medium (*i.e.*, [U^13^–C_6_]glucose or [U^13^–C_5_]glutamine), uptaken and metabolized into the cells, will lead to the production of molecules that contain ^13^C atoms at various positions. The compounds produced by the ^13^C tracer will be different only in the number of isotopic atoms incorporated, referred to as *mass isotopomers*. The MS generating ionized fragments, selectively detected by their mass-to-charge (m/z) ratio, allows to measure the Mass Isoptomer Distribution (MID) characterized as the fractional abundance of mass isotopomers, defined by M0, M1 to M*n*, *n* *∈* **N** (see Figure A1).

As an example of MID and the type of metabolic information that can be derived, we show the use of [U–^13^C_5_] glutamine tracers (Figure A1). In mammalian cells two major metabolic fates have been identified for glutamine: glutaminolysis and reductive carboxylation. In both pathways glutamine (Gln) ([U–^13^C_5_]glutamine) is deaminated to glutamate (Glu) generating M5 glutamate, which is then converted to *α*-ketoglutarate (M5 Akg) by glutamate dehydrogenase. At this point Akg can be oxidized into TCA cycle (forward TCA cycle), generating M4 labeled succinate (Succ), fumarate (Fum), malate (Mal), oxaloacetate (OAA) and citrate (Cit). On the other side, if Akg is metabolized through reductive carboxylation, we can observe labeling of M5 Cit which, exported from the mitochondria into the cytoplasm, will generate the labeling of acetyl-CoA (M2 AcCoA) and later lipids. Therefore, by exploiting appropriate tracers as ^13^C-glucose and ^15^N-glutamine, which are routinely used to assess specific metabolic activities in mammalian cells, we may know the overall contribution of nutrients in the biological system under investigation [257]. The use of stable isotope tracers not only improves the resolution of metabolomic techniques, but it also allows to perform MFA, which includes enzyme function quantification and metabolic flux reconstruction [248,255,257,258,259,260,261,262,263].

Several works have shown that ^13^C tracers provide a great amount of information regarding the dynamics of active metabolic pathways, as well as their participation in a specific metabolic pathway. As a result, the choice of selective tracer is extremely important, because it largely determines the precision with which one can estimate metabolic fluxes, especially in complex mammalian systems that require multiple substrates [256]. A very recent work has shown quantification and visualization *in vivo* of glucose metabolic flux in mouse brain after intraperitoneal injection of stable isotope-labeled glucose tracer ([^13^C_6_]glucose) [264], increasing the areas of application of metabolomics tools. Taken together, these evidences show the important role of metabolomics in the metabolic characterization of cell physiology and pathology.

## References

[1] Suthers P.F., Dasika M.S., Kumar V.S., Denisov G., Glass J.I., Maranas C.D. (2009). A genome-scale metabolic reconstruction of *Mycoplasma genitalium, iPS189*. PLoS Comput. Biol..

[2] Monk J.M., Charusanti P., Aziz R.K., Lerman J.A., Premyodhin N., Orth J.D., Feist A.M., Palsson B.Ø. (2013). Genome-scale metabolic reconstructions of multiple *Escherichia coli* strains highlight strain-specific adaptations to nutritional environments. Proc. Natl. Acad. Sci. USA.

[3] Heavner B.D., Smallbone K., Price N.D., Walker L.P. (2013). Version 6 of the consensus yeast metabolic network refines biochemical coverage and improves model performance. Database-Oxford.

[4] Thiele I., Swainston N., Fleming R.M.T., Hoppe A., Sahoo S., Aurich M.K., Haraldsdottir H., Mo M.L., Rolfsson O., Stobbe M.D. (2013). A community-driven global reconstruction of human metabolism. Nat. Biotechnol..

[5] Kubota K., Fukushima T., Yuji R., Miyano H., Hirayama K., Santa T., Imai K. (2005). Development of an HPLC-fluorescence determination method for carboxylic acids related to the tricarboxylic acid cycle as a metabolome tool. Biomed. Chromatogr..

[6] Nielsen J., Oliver S. (2005). The next wave in metabolome analysis. Trends Biotechnol..

[7] Griffin J.L. (2004). Metabolic profiles to define the genome: can we hear the phenotypes?. Philos. Trans. R. Soc. B.

[8] Griffin J.L., Shockcor J.P. (2004). Metabolic profiles of cancer cells. Nat. Rev. Cancer.

[9] Patton A.L., Seely K.A., Chimalakonda K.C., Tran J.P., Trass M., Miranda A., Fantegrossi W.E., Kennedy P.D., Dobrowolski P., Radominska-Pandya A. (2013). Targeted metabolomic approach for assessing human synthetic cannabinoid exposure and pharmacology. Anal. Chem..

[10] Robertson D.G., Watkins P.B., Reily M.D. (2011). Metabolomics in toxicology: preclinical and clinical applications. Toxicol. Sci..

[11] Roessner U., Luedemann A., Brust D., Fiehn O., Linke T., Willmitzer L., Fernie A.R. (2001). Metabolic profiling allows comprehensive phenotyping of genetically or environmentally modified plant systems. Plant Cell.

[12] Nordström A., Lewensohn R. (2010). Metabolomics: Moving to the clinic. J. Neuroimmune Pharm..

[13] Alberghina L., Gaglio D., Moresco R.M., Gilardi M.C., Messa C., Vanoni M. (2014). A systems biology road map for the discovery of drugs targeting cancer cell metabolism. Curr. Pharm. Design.

[14] Ward P.S., Thompson C.B. (2012). Metabolic reprogramming: A cancer hallmark even Warburg did not anticipate. Cancer Cell.

[15] Hanahan D., Weinberg R.A. (2011). Hallmarks of cancer: The next generation. Cell.

[16] Senn T., Hazen S.L., Tang W. (2012). Translating metabolomics to cardiovascular biomarkers. Prog. Cardiovasc. Dis..

[17] Trushina E., Mielke M.M. (2014). Recent advances in the application of metabolomics to Alzheimer’s disease. BBA-Mol. Basis Dis..

[18] Vermeersch K.A., Styczynski M.P. (2013). Applications of metabolomics in cancer research. J. Carcinog..

[19] Toya Y., Shimizu H. (2013). Flux analysis and metabolomics for systematic metabolic engineering of microorganisms. Biotechnol. Adv..

[20] Feng X., Page L., Rubens J., Chircus L., Colletti P., Pakrasi H.B., Tang Y.J. (2011). Bridging the gap between fluxomics and industrial biotechnology. Biomed. Res. Int..

[21] Gianchandani E.P., Chavali A.K., Papin J.A. (2010). The application of flux balance analysis in systems biology. WIREs Syst. Biol. Med..

[22] Alberghina L., Westerhoff H.V. (2005). Systems Biology: Definitions and Perspectives.

[23] Stelling J. (2004). Mathematical models in microbial systems biology. Curr. Opin. Microbiol..

[24] Wang L., Hatzimanikatis V. (2006). Metabolic engineering under uncertainty. I: Framework development. Metab. Eng..

[25] Steuer R., Gross T., Selbig J., Blasius B. (2006). Structural kinetic modeling of metabolic networks. Proc. Natl. Acad. Sci. USA.

[26] Murabito E., Smallbone K., Swinton J., Westerhoff H.V., Steuer R. (2011). A probabilistic approach to identify putative drug targets in biochemical networks. J. R. Soc. Interface.

[27] Jeong H., Tombor B., Albert R., Oltvai Z.N., Barabási A.L. (2000). The large-scale organization of metabolic networks. Nature.

[28] Hopkins A.L. (2008). Network pharmacology: the next paradigm in drug discovery. Nat. Chem. Biol..

[29] Feist A.M., Herrgård M.J., Thiele I., Reed J.L., Palsson B.Ø. (2008). Reconstruction of biochemical networks in microorganisms. Nat. Rev. Microbiol..

[30] Karp P.D., Riley M., Paley S.M., Pelligrini-Toole A. (1996). EcoCyc: An encyclopedia of *Escherichia coli* genes and metabolism. Nucleic Acids Res..

[31] Karp P.D., Ouzounis C.A., Paley S.M. HinCyc: A knowledge base of the complete genome and metabolic pathways of *H. influenzae*. Proceedings of the International Conference on Intelligent Systems for Molecular Biology (ISMB).

[32] Kanehisa M., Goto S. (2000). KEGG: Kyoto Encyclopedia of Genes and Genomes. Nucleic Acids Res..

[33] Levchenko A. (2003). Dynamical and integrative cell signaling: Challenges for the new biology. Biotechnol. Bioeng..

[34] Soon W.W., Hariharan M., Snyder M.P. (2013). High-throughput sequencing for biology and medicine. Mol. Syst. Biol..

[35] Hyduke D.R., Lewis N.E., Palsson B.Ø. (2013). Analysis of omics data with genome-scale models of metabolism. Mol. Biosyst..

[36] Edwards J.S., Palsson B.Ø. (1999). Systems properties of the *Haemophilus influenzae* Rd metabolic genotype. J. Biol. Chem..

[37] Knoop H., Gründel M., Zilliges Y., Lehmann R., Hoffmann S., Lockau W., Steuer R. (2013). Flux balance analysis of cyanobacterial metabolism: The metabolic network of *Synechocystis* sp. PCC 6803. PLoS Comput. Biol..

[38] Xu N., Liu L., Zou W., Liu J., Hua Q., Chen J. (2013). Reconstruction and analysis of the genome-scale metabolic network of *Candida glabrata*. Mol. Biosyst..

[39] Duarte N.C., Becker S.A., Jamshidi N., Thiele I., Mo M.L., Vo T.D., Srivas R., Palsson B.Ø. (2007). Global reconstruction of the human metabolic network based on genomic and bibliomic data. Proc. Natl. Acad. Sci. USA.

[40] Monaco M.K., Sen T.Z., Dharmawardhana P.D., Ren L., Schaeffer M., Naithani S., Ama-rasinghe V., Thomason J., Harper L., Gardiner J. (2013). Maize metabolic network construc- tion and transcriptome analysis. Plant Genome.

[41] McCloskey D., Palsson B.Ø., Feist A.M. (2013). Basic and applied uses of genome-scale metabolic network reconstructions of *Escherichia coli*. Mol. Syst. Biol..

[42] Thiele I., Palsson B.Ø. (2010). A protocol for generating a high-quality genome-scale metabolic reconstruction. Nat. Protoc..

[43] Herrgård M.J., Swainston N., Dobson P., Dunn W.B., Arga K.Y., Arvas M., Blüthgen N., Borger S., Costenoble R., Heinemann M. (2008). A consensus yeast metabolic network reconstruction obtained from a community approach to systems biology. Nat. Biotechnol..

[44] Devoid S., Overbeek R., deJongh M., Vonstein V., Best A.A., Henry C. (2013). Automated genome annotation and metabolic model reconstruction in the SEED. Systems Metabolic Engineering.

[45] Kumar V.S., Dasika M.S., Maranas C.D. (2007). Optimization based automated curation of metabolic reconstructions. BMC Bioinform..

[46] Henry C.S., DeJongh M., Best A.A., Frybarger P.M., Linsay B., Stevens R.L. (2010). High-throughput generation, optimization and analysis of genome-scale metabolic models. Nat. Biotechnol..

[47] Latendresse M., Krummenacker M., Trupp M., Karp P.D. (2012). Construction and completion of flux balance models from pathway databases. Bioinformatics.

[48] Latendresse M. (2014). Efficiently gap-filling reaction networks. BMC Bioinform..

[49] Thorleifsson S.G., Thiele I. (2011). rBioNet: A COBRA toolbox extension for reconstructing high-quality biochemical networks. Bioinformatics.

[50] Stobbe M.D., Houten S.M., Jansen G.A., van Kampen A.H.C., Moerland P.D. (2011). Critical assessment of human metabolic pathway databases: a stepping stone for future integration. BMC Syst. Biol..

[51] Stobbe M.D., Swertz M.A., Thiele I., Rengaw T., van Kampen A.H.C., Moerland P.D. (2013). Consensus and conflict cards for metabolic pathway databases. BMC Syst. Biol..

[52] Agren R., Bordel S., Mardinoglu A., Pornputtapong N., Nookaew I., Nielsen J. (2012). Reconstruction of genome-scale active metabolic networks for 69 human cell types and 16 cancer types using INIT. PLoS Comput. Biol..

[53] Uhlen M., Oksvold P., Fagerberg L., Lundberg E., Jonasson K., Forsberg M., Zwahlen M., Kampf C., Wester K., Hober S. (2010). Towards a knowledge-based Human Protein Atlas. Nat. Biotechnol..

[54] Monk J., Nogales J., Palsson B.Ø. (2014). Optimizing genome-scale network reconstructions. Nat. Biotechnol..

[55] Schellenberger J., Lewis N.E., Palsson B.Ø. (2011). Elimination of thermodynamically infeasible loops in steady-state metabolic models. Biophys. J..

[56] De Martino D., Capuani F., Mori M., De Martino A., Marinari E. (2013). Counting and correcting thermodynamically infeasible flux cycles in genome-scale metabolic networks. Metabolites.

[57] Otero J.M., Cimini D., Patil K.R., Poulsen S.G., Olsson L., Nielsen J. (2013). Industrial systems biology of *Saccharomyces cerevisiae* enables novel succinic acid cell factory. PLoS One.

[58] Agren R., Otero J.M., Nielsen J. (2013). Genome-scale modeling enables metabolic engineering of *Saccharomyces cerevisiae* for succinic acid production. J. Ind. Microbiol. Biotechnol..

[59] Oberhardt M.A., Palsson B.Ø., Papin J.A. (2009). Applications of genome-scale metabolic reconstructions. Mol. Syst. Biol..

[60] Molenaar D., van Berlo R., de Ridder D., Teusink B. (2009). Shifts in growth strategies reflect tradeoffs in cellular economics. Mol. Syst. Biol..

[61] Vazquez A., Liu J., Zhou Y., Oltvai Z.N. (2010). Catabolic efficiency of aerobic glycolysis: The Warburg effect revisited. BMC Syst. Biol..

[62] Teusink B., Passarge J., Reijenga C.A., Esgalhado E., van der Weijden C.C., Schepper M., Walsh M.C., Bakker B.M., van Dam K., Westerhoff H.V. (2000). Can yeast glycolysis be understood in terms of *in vitro* kinetics of the constituent enzymes? Testing biochemistry. Eur. J. Biochem..

[63] Smallbone K., Messiha H.L., Carroll K.M., Winder C.L., Malys N., Dunn W.B., Murabito E., Swainston N., Dada J.O., Khan F. (2013). A model of yeast glycolysis based on a consistent kinetic characterisation of all its enzymes. FEBS Lett..

[64] Kerkhoven E.J., Achcar F., Alibu V.P., Burchmore R.J., Gilbert I.H., Trybiło M., Driessen N.N., Gilbert D., Breitling R., Bakker B.M. (2013). Handling uncertainty in dynamic models: The pentose phosphate pathway in *Trypanosoma brucei*. PLoS Comput. Biol..

[65] Damiani C., Pescini D., Colombo R., Molinari S., Alberghina L., Vanoni M., Mauri G. (2014). An ensemble evolutionary constraint-based approach to understand the emergence of metabolic phenotypes. Nat. Comput..

[66] Diaz-Ruiz R., Rigoulet M., Devin A. (2011). The Warburg and Crabtree effects: On the origin of cancer cell energy metabolism and of yeast glucose repression. BBA-Bioenergetics.

[67] Resendis-Antonio O., Checa A., Encarnación S. (2010). Modeling core metabolism in cancer cells: Surveying the topology underlying the Warburg effect. PLoS One.

[68] Mogilner A., Wollman R., Marshall W.F. (2006). Quantitative modeling in cell biology: What is it good for?. Dev. Cell.

[69] Ingolia N.T., Murray A.W. (2004). The ups and downs of modeling the cell cycle. Curr. Biol..

[70] Moles C.G., Mendes P., Banga J.R. (2003). Parameter estimation in biochemical pathways: A comparison of global optimization methods. Genome Res..

[71] Nobile M.S., Besozzi D., Cazzaniga P., Mauri G., Pescini D., Giacobini M., Vanneschi L., Bush W. (2012). A GPU-based multi-swarm PSO method for parameter estimation in stochastic biological systems exploiting discrete-time target series. Evolutionary Computation Machine Learning and Data Mining in Bioinformatics.

[72] Guillén-Gosálbez G., Miró A., Alves R., Sorribas A., Jiménez L. (2013). Identification of regulatory structure and kinetic parameters of biochemical networks via mixed-integer dynamic optimization. BMC Syst. Biol..

[73] Hendrickx D.M., Hendriks M.M.W.B., Eilers P.H.C., Smilde A.K., Hoefsloot H.C.J. (2011). Reverse engineering of metabolic networks, a critical assessment. Mol. Biosyst..

[74] Quach M., Brunel N., d’Alché Buc F. (2007). Estimating parameters and hidden variables in non-linear state-space models based on ODEs for biological networks inference. Bioinformatics.

[75] Raue A., Becker V., Klingmüller U., Timmer J. (2010). Identifiability and observability analysis for experimental design in nonlinear dynamical models. Chaos.

[76] Dräger A., Kronfeld M., Ziller M.J., Supper J., Planatscher H., Magnus J.B., Oldiges M., Kohlbacher O., Zell A. (2009). Modeling metabolic networks in *C. glutamicum*: A comparison of rate laws in combination with various parameter optimization strategies. BMC Syst. Biol..

[77] Caspi R., Altman T., Dreher K., Fulcher C.A., Subhraveti P., Keseler I.M., Kothari A., Krummenacker M., Latendresse M., Mueller L.A. (2012). The MetaCyc database of metabolic pathways and enzymes and the BioCyc collection of pathway/genome databases. Nucleic Acids Res..

[78] Dash R.K., Li Y., Kim J., Saidel G.M., Cabrera M.E. (2008). Modeling cellular metabolism and energetics in skeletal muscle: large-scale parameter estimation and sensitivity analysis. IEEE Trans. Bio-Med. Eng..

[79] Lasdon L., Waren A., Jain A., Ratner M. (1978). Design and testing of a generalized reduced gradient code for nonlinear programming. ACM Trans. Math. Software.

[80] Bazil J.N., Buzzard G.T., Rundell A.E. (2010). Modeling mitochondrial bioenergetics with integrated volume dynamics. PLoS Comput. Biol..

[81] Kirkpatrick S., Gelatt C.D., Vecchi M.P. (1983). Optimization by simulated annealing. Science.

[82] Eberhart R.C., Kennedy J. A new optimiser using particle swarm theory. Proceedings of the IEEE Sixth International Symposium on Micro Machine and Human Science.

[83] Storn R. On the usage of differential evolution for function optimization. Proceedings of the Biennial Conference of the North American Fuzzy Information Processing Society.

[84] Besozzi D., Cazzaniga P., Mauri G., Pescini D., Vanneschi L., Pizzuti C., Ritchie M.D., Giacobini M. (2009). A compar-ison of genetic algorithms and particle swarm optimization for parameter estimation in stochastic biochemical systems. Evolutionary Computation, Machine Learn-ing and Data Mining in Bioinformatics.

[85] Clerc M. (2010). Particle Swarm Optimization.

[86] Mendes P., Kell D.B. (1998). Non-linear optimization of biochemical pathways: Applications to metabolic engineering and parameter estimation. Bioinformatics.

[87] Marquardt D.W. (1963). An algorithm for least-squares estimation of nonlinear parameters. J. Soc. Ind. Appl. Math..

[88] Villaverde A.F., Egea J.A., Banga J.R. (2012). A cooperative strategy for parameter estimation in large scale systems biology models. BMC Syst. Biol..

[89] Koza J.R., Mydlowec W., Lanza G., Yu J., Keane M.A. Reverse engineering of metabolic pathways from observed data using genetic programming. Proceedings of the IEEE Pacific Symposium on Biocomputing.

[90] Koza J.R. (1992). Genetic Programming: On the Programming of Computers by Means of Natural Selection.

[91] Sugimoto M., Kikuchi S., Tomita M. (2005). Reverse engineering of biochemical equations from time-course data by means of genetic programming. Biosystems.

[92] Cho D., Cho K., Zhang B. (2006). Identification of biochemical networks by S-tree based genetic programming. Bioinformatics.

[93] Nobile M.S., Besozzi D., Cazzaniga P., Pescini D., Mauri G. Reverse engineering of kinetic reaction networks by means of cartesian genetic programming and particle swarm optimization. Proceedings of the IEEE Congress on Evolutionary Computation (CEC).

[94] Miller J., Thomson P. Cartesian Genetic Programming. Proceedings of the Third European Conference on Genetic Programming (EuroGP2000).

[95] Szederkenyi G., Banga J.R., Alonso A.A. (2011). Inference of complex biological networks: distinguishability issues and optimization-based solutions. BMC Syst. Biol..

[96] Çakır T., Hendriks M.M.W.B., Westerhuis J.A., Smilde A.K. (2009). Metabolic network discovery through reverse engineering of metabolome data. Metabolomics.

[97] Arkin A., Ross J. (1995). Statistical construction of chemical reaction mechanisms from measured time-series. J. Phys. Chem..

[98] Damiani C., Lecca P. Model identification using correlation-based inference and transfer entropy estimation. Proceedings of the IEEE Fifth UKSim European Symposium on Computer Modeling and Simulation (EMS).

[99] Schreiber T. (2000). Measuring information transfer. Phys. Rev. Lett..

[100] Vance W., Arkin A., Ross J. (2002). Determination of causal connectivities of species in reaction networks. Proc. Natl. Acad. Sci. USA.

[101] Tran L.M., Rizk M.L., Liao J.C. (2008). Ensemble modeling of metabolic networks. Biophys. J..

[102] Jia G., Stephanopoulos G., Gunawan R. (2012). Ensemble kinetic modeling of metabolic networks from dynamic metabolic profiles. Metabolites.

[103] Henry C.S., Broadbelt L.J., Hatzimanikatis V. (2007). Thermodynamics-based metabolic flux analysis. Biophys. J..

[104] Miškovic´ L., Hatzimanikatis V. (2011). Modeling of uncertainties in biochemical reactions. Biotechnol. Bioeng..

[105] Link H., Kochanowski K., Sauer U. (2013). Systematic identification of allosteric protein-metabolite interactions that control enzyme activity *in vivo*. Nat. Biotechnol..

[106] Zomorrodi A.R., Lafontaine Rivera J.G., Liao J.C., Maranas C.D. (2013). Optimization-driven identification of genetic perturbations accelerates the convergence of model parameters in ensemble modeling of metabolic networks. Biotechnol. J..

[107] Link H., Christodoulou D., Sauer U. (2014). Advancing metabolic models with kinetic information. Curr. Opin. Biotech..

[108] Bollobás B. (1998). Modern Graph Theory.

[109] Aittokallio T., Schwikowski B. (2006). Graph-based methods for analysing networks in cell biology. Brief Bioinform..

[110] Albert R. (2005). Scale-free networks in cell biology. J. Cell Sci..

[111] Caldarelli G. (2007). Scale-Free Networks. Complex Webs in Nature and Technology.

[112] Romero P., Karp P.D. Nutrition-related analysis of pathway/genome databases. Proceedings of the Pacific Symposium on Biocomputing.

[113] Light S., Kraulis P., Elofsson A. (2005). Preferential attachment in the evolution of metabolic networks. BMC Genomics.

[114] Hordijk W., Steel M. (2004). Detecting autocatalytic, self-sustaining sets in chemical reaction systems. J. Theor. Biol..

[115] Albert R., Jeong H., Barabási A.L. (2000). Error and attack tolerance of complex networks. Nature.

[116] Fell D.A., Wagner A. (2000). The small world of metabolism. Nat. Biotechnol..

[117] Wagner A., Fell D.A. (2001). The small world inside large metabolic networks. Proc. R. Soc. B-Biol. Sci..

[118] 118. Csete M.E., Doyle J.C. (2004). Bow ties, metabolism and disease. Trends Biotechnol..

[119] Zhao J., Yu H., Luo J.H., Cao Z.W., Li Y.X. (2006). Hierarchical modularity of nested bow-ties in metabolic networks. BMC Bioinform..

[120] Resendis-Antonio O., Hernández M., Mora Y., Encarnación S. (2012). Functional modules, structural topology, and optimal activity in metabolic networks. PLoS Comput. Biol..

[121] Montañez R., Medina M.A., Solé R.V., Rodríguez-Caso C. (2010). When metabolism meets topology: Reconciling metabolite and reaction networks. Bioessays.

[122] Ravasz E., Somera A.L., Mongru D.A., Oltvai Z.N., Barabási A.L. (2002). Hierarchical organization of modularity in metabolic networks. Science.

[123] Chen M., Hofestädt R. (2003). Quantitative Petri net model of gene regulated metabolic networks in the cell. In Silico Biol..

[124] Reddy V.N., Mavrovouniotis M.L., Liebman M.N. Petri net representations in metabolic pathways. Proceedings of the ISMB.

[125] Zevedei-Oancea I., Schuster S. (2003). Topological analysis of metabolic networks based on Petri net theory. In Silico Biol..

[126] Lewis N.E., Nagarajan H., Palsson B.Ø. (2012). Constraining the metabolic genotype–phenotype relationship using a phylogeny of *in silico* methods. Nat. Rev. Microbiol..

[127] COBRA Methods. http://cobramethods.wikidot.com/methods.

[128] Orth J.D., Thiele I., Palsson B.Ø. (2010). What is flux balance analysis?. Nat. Biotechnol..

[129] Lee J.M., Gianchandani E.P., Papin J.A. (2006). Flux balance analysis in the era of metabolomics. Brief Bioinform..

[130] Simeonidis E., Murabito E., Smallbone K., Westerhoff H.V. (2010). Why does yeast ferment? A flux balance analysis study. Biochem. Soc. T.

[131] Bro C., Regenberg B., Förster J., Nielsen J. (2006). *In silico* aided metabolic engineering of *Saccharomyces cerevisiae* for improved bioethanol production. Metab. Eng..

[132] Burgard A.P., Maranas C.D. (2003). Optimization-based framework for inferring and testing hypothesized metabolic objective functions. Biotechnol. Bioeng..

[133] Ramakrishna R., Edwards J.S., McCulloch A., Palsson B.Ø. (2001). Flux-balance analysis of mitochondrial energy metabolism: consequences of systemic stoichiometric constraints. Am. J. Physiol.-Reg. I.

[134] Schuster S., Hilgetag C. (1994). On elementary flux modes in biochemical reaction systems at steady state. J. Biol. Syst..

[135] Schilling C.H., Letscher D., Palsson B.Ø. (2000). Theory for the systemic definition of metabolic pathways and their use in interpreting metabolic function from a pathway-oriented perspective. J. Theor. Biol..

[136] Hunt K.A., Folsom J.P., Taffs R.L., Carlson R.P. (2014). Complete enumeration of elementary flux modes through scalable, demand-based subnetwork definition. Bioinformatics.

[137] De Figueiredo L.F., Podhorski A., Rubio A., Kaleta C., Beasley J.E., Schuster S., Planes F.J. (2009). Computing the shortest elementary flux modes in genome-scale metabolic networks. Bioinformatics.

[138] Mahadevan R., Schilling C.H. (2003). The effects of alternate optimal solutions in constraint-based genome-scale metabolic models. Metab. Eng..

[139] Gudmundsson S., Thiele I. (2010). Computationally efficient flux variability analysis. BMC Bioinform..

[140] Feist A., Palsson B.Ø. (2010). The biomass objective function. Curr. Opin. Microbiol..

[141] Schellenberger J., Palsson B.Ø. (2009). Use of randomized sampling for analysis of metabolic networks. J. Biol. Chem..

[142] Mahadevan R., Edwards J.S., Doyle III F.J. (2002). Dynamic flux balance analysis of diauxic growth in *Escherichia coli*. Biophys. J..

[143] Covert M.W., Schilling C.H., Palsson B.Ø. (2001). Regulation of gene expression in flux balance models of metabolism. J. Theor. Biol..

[144] Herrgård M.J., Lee B.S., Portnoy V., Palsson B.Ø. (2006). Integrated analysis of regulatory and metabolic networks reveals novel regulatory mechanisms in *Saccharomyces cerevisiae*. Genome Res..

[145] Lee J.M., Gianchandani E.P., Eddy J.A., Papin J.A. (2008). Dynamic analysis of integrated signaling, metabolic, and regulatory networks. PLoS Comput. Biol..

[146] Machado D., Herrgård M. (2014). Systematic evaluation of methods for integration of transcriptomic data into constraint-based models of metabolism. PLoS Comput. Biol..

[147] Burgard A.P., Nikolaev E.V., Schilling C.H., Maranas C.D. (2004). Flux coupling analysis of genome-scale metabolic network reconstructions. Genome Res..

[148] Papin J.A., Reed J.L., Palsson B.Ø. (2004). Hierarchical thinking in network biology: the unbiased modularization of biochemical networks. Trends Biochem. Sci..

[149] Reed J.L., Palsson B.Ø. (2004). Genome-scale in silico models of *E. coli* have multiple equivalent phenotypic states: Assessment of correlated reaction subsets that comprise network states. Genome Res..

[150] Petzold L.R. (1983). Automatic selection of methods for solving stiff and nonstiff systems of ordinary differential equations. SIAM J. Sci. Stat. Comput..

[151] Brown P.N., Byrne G.D., Hindmarsh A.C. (1989). VODE: A variable-coefficient ODE solver. SIAM J. Sci. Stat. Comput..

[152] Metelkin E., Goryanin I., Demin O. (2006). Mathematical modeling of mitochondrial adenine nucleotide translocase. Biophys. J..

[153] Guillaud F., Dröse S., Kowald A., Brandt U., Klipp E. (2014). Superoxide production by cytochrome *bc*1 complex: A mathematical model. BBA-Bioenergetics.

[154] Chang I., Heiske M., Letellier T., Wallace D., Baldi P. (2011). Modeling of mitochondria bioenergetics using a composable chemiosmotic energy transduction rate law: Theory and experimental validation. PLoS One.

[155] Korzeniewski B., Zoladz J.A. (2001). A model of oxidative phosphorylation in mammalian skeletal muscle. Biophys. Chem..

[156] Beard D.A. (2005). A biophysical model of the mitochondrial respiratory system and oxidative phosphorylation. PLoS Comput. Biol..

[157] König M., Bulik S., Holzhütter H.G. (2012). Quantifying the contribution of the liver to glucose homeostasis: A detailed kinetic model of human hepatic glucose metabolism. PLoS Comput. Biol..

[158] Reed M.C., Thomas R.L., Pavisic J., James S.J., Ulrich C.M., Nijhout H.F. (2008). A mathematical model of glutathione metabolism. Theor. Biol. Med. Model..

[159] Achcar F., Kerkhoven E.J., Bakker B.M., Barrett M.P., Breitling R., The SilicoTryp Consortium (2012). Dynamic modelling under uncertainty: The case of *Trypanosoma brucei* energy metabolism. PLoS Comput. Biol..

[160] Kinsey S.T., Locke B.R., Dillaman R.M. (2011). Molecules in motion: influences of diffusion on metabolic structure and function in skeletal muscle. J. Exp. Biol..

[161] Baras F., Mansour M.M. (1996). Reaction-diffusion master equation: A comparison with microscopic simulations. Phys. Rev. E.

[162] Bernstein D. (2005). Simulating mesoscopic reaction-diffusion systems using the Gillespie algorithm. Phys. Rev. E.

[163] Gruenert G., Ibrahim B., Lenser T., Lohel M., Hinze T., Dittrich P. (2010). Rule-based spatial modeling with diffusing, geometrically constrained molecules. BMC Bioinform..

[164] Zhou L., Aon M., Almas T., Cortassa S., Winslow R., O’Rourke B. (2010). A reaction-diffusion model of ROS-induced ROS release in a mitochondrial network. PLoS Comput. Biol..

[165] Dasika S.K., Kinsey S.T., Locke B.R. (2012). Facilitated diffusion of myoglobin and creatine kinase and reaction–diffusion constraints of aerobic metabolism under steady-state conditions in skeletal muscle. Biotechnol. Bioeng..

[166] Yugi K., Nakayama Y., Kinoshita A., Tomita M. (2005). Hybrid dynamic/static method for large-scale simulation of metabolism. Theor. Biol. Med. Model..

[167] Ishii N., Nakayama Y., Tomita M. (2007). Distinguishing enzymes using metabolome data for the hybrid dynamic/static method. Theor. Biol. Med. Model..

[168] Osana Y., Fukushima T., Yoshimi M. An FPGA-based multi-model simulation method for biochemical systems. Proceedings of the 19th IEEE International Parallel and Distributed Processing Symposium.

[169] Li H., Cao H., Petzold L.R., Gillespie D.T. (2008). Algorithms and software for stochastic simulation of biochemical reacting systems. Biotechnol. Prog..

[170] Nobile M.S., Cazzaniga P., Besozzi D., Pescini D., Mauri G. (2014). cuTauLeaping: A GPU-powered tau-leaping stochastic simulator for massive parallel analyses of biological systems. PLoS One.

[171] Nobile M.S., Besozzi D., Cazzaniga P., Mauri G. (2014). GPU-accelerated simulations of mass-action kinetics models with cupSODA. J. Supercomput..

[172] Kiviet D.J., Nghe P., Walker N., Boulineau S., Sunderlikova V., Tans S.J. (2014). Stochasticity of metabolism and growth at the single-cell level. Nature.

[173] Andersen M.R., Nielsen M.L., Nielsen J. (2008). Metabolic model integration of the bibliome, genome, metabolome and reactome of *Aspergillus niger*. Mol. Syst. Biol..

[174] Varma A., Palsson B.Ø. (1994). Stoichiometric flux balance models quantitatively predict growth and metabolic by-product secretion in wild-type *Escherichia coli* W3110. Appl. Environ. Microbiol..

[175] Edwards J., Palsson B.Ø. (2000). The *Escherichia coli* MG1655 in silico metabolic genotype: its definition, characteristics, and capabilities. Proc. Natl. Acad. Sci. USA.

[176] Förster J., Famili I., Palsson B.Ø., Nielsen J. (2003). Large-scale evaluation of *in silico* gene deletions in *Saccharomyces cerevisiae*. OMICS.

[177] Saltelli A., Ratto M., Andres T., Campolongo F., Cariboni J., Gatelli D., Saisana M., Tarantola S. (2008). Global Sensitivity Analysis: The Primer.

[178] Saltelli A., Tarantola S., Campolongo F. (2000). Sensitivity anaysis as an ingredient of modeling. Stat. Sci..

[179] Degenring D., Froemel C., Dikta G., Takors R. (2004). Sensitivity analysis for the reduction of complex metabolism models. J. Process Contr..

[180] Fell D.A. (1992). Metabolic control analysis: A survey of its theoretical and experimental development. Biochem. J..

[181] Kohn M.C. (1983). Computer simulation of metabolism in palmitate-perfused rat heart. III. Sensitivity analysis. Ann. Biomed. Eng..

[182] Zi Z. (2011). Sensitivity analysis approaches applied to systems biology models. IET Syst. Biol..

[183] Oshiro M., Shinto H., Tashiro Y., Miwa N., Sekiguchi T., Okamoto M., Ishizaki A., Sonomoto K. (2009). Kinetic modeling and sensitivity analysis of xylose metabolism in *Lactococcus lactis* IO-1. J. Biosci. Bioeng..

[184] Shinto H., Tashiro Y., Yamashita M., Kobayashi G., Sekiguchi T., Hanai T., Kuriya Y., Okamoto M., Sonomoto K. (2007). Kinetic modeling and sensitivity analysis of acetone-butanol-ethanol production. J. Biotechnol..

[185] Chan K., Saltelli A., Tarantola S. Sensitivity analysis of model output: Variance-based methods make the difference. Proceedings of the 29th conference on Winter simulation.

[186] Di Maggio J., Diaz Ricci J.C., Diaz M.S. (2010). Global sensitivity analysis in dynamic metabolic networks. Comput. Chem. Eng..

[187] Helton J.C., Davis F.J. (2002). Illustration of sampling-based methods for uncertainty and sensitivity analysis. Risk Anal..

[188] Damiani C., Filisetti A., Graudenzi A., Lecca P. (2013). Parameter sensitivity analysis of stochastic models: Application to catalytic reaction networks. Comput. Biol. Chem..

[189] Kareva I. (2011). Prisoner’s dilemma in cancer metabolism. PLoS One.

[190] Sajitz-Hermstein M., Nikoloski Z. (2012). Restricted cooperative games on metabolic networks reveal functionally important reactions. J. Theor. Biol..

[191] Yang R., Lenaghan S.C., Wikswo J.P., Zhang M. (2011). External control of the GAL network in *S. cerevisiae*: A view from control theory. PLoS One.

[192] Palumbo P., Mavelli G., Farina L., Alberghina L. (2010). Networks and circuits in cell regulation. Biochem. Biophys. Res. Commun..

[193] Hinze T., Schumann M., Bodenstein C., Heiland I., Schuster S. (2011). Biochemical frequency control by synchronisation of coupled Repressilators: An *in silico* study of modules for circadian clock systems. Comput. Intell. Neurosci..

[194] Broom M., Rychtáˇr J. (2013). Game-Theoretical Models in Biology.

[195] Cosentino C., Bates D. (2012). Feedback Control in Systems Biology.

[196] Sontag E.D. (2004). Some new directions in control theory inspired by systems biology. Syst. Biol..

[197] Csete M.E., Doyle J.C. (2002). Reverse engineering of biological complexity. Science.

[198] Wellstead P., Bullinger E., Kalamatianos D., Mason O., Verwoerd M. (2008). The rôle of control and system theory in systems biology. Annu. Rev. Control.

[199] Szallasi Z., Stelling J., Periwal V. (2006). Systems Modeling in Cellular Biology.

[200] Cloutier M., Wellstead P. (2010). The control systems structures of energy metabolism. J. R. Soc. Interface.

[201] Wellstead P., Cloutier M. (2011). An energy systems approach to Parkinson’s disease. WIREs Syst. Biol. Med..

[202] Federowicz S., Kim D., Ebrahim A., Lerman J., Nagarajan H., Cho B.K., Zengler K., Palsson B.Ø. (2014). Determining the control circuitry of redox metabolism at the genome-scale. PLoS Genet..

[203] Reeves G.T., Fraser S.E. (2009). Biological systems from an engineer’s point of view. PLoS Biol..

[204] Karr J.R., Sanghvi J.C., Macklin D.N., Gutschow M.V., Jacobs J.M., Bolival B., Assad-Garcia N., Glass J.I., Covert M.W. (2012). A whole-cell computational model predicts phenotype from genotype. Cell.

[205] Vander Heiden M.G., Cantley L.C., Thompson C.B. (2009). Understanding the Warburg effect: The metabolic requirements of cell proliferation. Science.

[206] Shlomi T., Benyamini T., Gottlieb E., Sharan R., Ruppin E. (2011). Genome-scale metabolic modeling elucidates the role of proliferative adaptation in causing the Warburg effect. PLoS Comput. Biol..

[207] Yizhak K., Le Dévédec S.E., Rogkoti V.M., Baenke F., Boer V.C., Frezza C., Schulze A., Water B., Ruppin E. (2014). A computational study of the Warburg effect identifies metabolic targets inhibiting cancer migration. Mol. Syst. Biol..

[208] Folger O., Jerby L., Frezza C., Gottlieb E., Ruppin E., Shlomi T. (2011). Predicting selective drug targets in cancer through metabolic networks. Mol. Syst. Biol..

[209] Frezza C., Zheng L., Folger O., Rajagopalan K., MacKenzie E., Jerby L., Micaroni M., Chaneton B., Adam J., Hedley A. (2011). Haem oxygenase is synthetically lethal with the tumour suppressor fumarate hydratase. Nature.

[210] Nam H., Campodonico M., Bordbar A., Hyduke D.R., Kim S., Zielinski D.C., Bernhard O.P. (2014). A systems approach to predict oncometabolites via context-specific genome-scale metabolic networks. PLoS Comput. Biol..

[211] Brynildsen M.P., Winkler J.A., Spina C.S., MacDonald I.C., Collins J.J. (2013). Potentiating antibacterial activity by predictably enhancing endogenous microbial ROS production. Nat. Biotechnol..

[212] Kim H.U., Kim S.Y., Jeong H., Kim T.Y., Kim J.J., Choy H.E., Yi K.Y., Rhee J.H., Lee S.Y. (2011). Integrative genome-scale metabolic analysis of Vibrio vulnificus for drug targeting and discovery. Mol. Syst. Biol..

[213] Van Heerden J.H., Wortel M.T., Bruggeman F.J., Heijnen J.J., Bollen Y.J.M., Planqué R., Hulshof J., O’Toole T.G., Wahl S.A., Teusink B. (2014). Lost in transition: Start-up of glycolysis yields subpopulations of nongrowing cells. Science.

[214] Khazaei T., McGuigan A., Mahadevan R. (2012). Ensemble modeling of cancer metabolism. Front Physiol..

[215] Nielsen J. (2001). Metabolic engineering. Appl. Microbiol. Biotech..

[216] Bordbar A., Monk J.M., King Z.A., Palsson B.Ø. (2014). Constraint-based models predict metabolic and associated cellular functions. Nat. Rev. Genet..

[217] Edwards J.S., Ibarra R.U., Palsson B.Ø. (2001). *In silico* predictions of *Escherichia coli* metabolic capabilities are consistent with experimental data. Nat. Biotechnol..

[218] Lee S.Y., Lee D.Y., Kim T.Y. (2005). Systems biotechnology for strain improvement. Trends Biotechnol..

[219] Yim H., Haselbeck R., Niu W., Pujol-Baxley C., Burgard A., Boldt J., Khandurina J., Trawick J.D., Osterhout R.E., Stephen R. (2011). Metabolic engineering of *Escherichia coli* for direct production of 1, 4-butanediol. Nat. Chem. Biol..

[220] Gille C., Bölling C., Hoppe A., Bulik S., Hoffmann S., Hübner K., Karlstädt A., Ganeshan R., König M., Rother K. (2010). HepatoNet1: A comprehensive metabolic reconstruction of the human hepatocyte for the analysis of liver physiology. Mol. Syst. Biol..

[221] Hao T., Ma H., Zhao X., Goryanin I. (2010). Compartmentalization of the Edinburgh human metabolic network. BMC Bioinform..

[222] Österlund T., Nookaew I., Bordel S., Nielsen J. (2013). Mapping condition-dependent regulation of metabolism in yeast through genome-scale modeling. BMC Syst. Biol..

[223] Sahoo S., Franzson L., Jonsson J.J., Thiele I. (2012). A compendium of inborn errors of metabolism mapped onto the human metabolic network. Mol. Biosyst..

[224] Yugi K. (2013). Dynamic kinetic modeling of mitochondrial energy metabolism. E-Cell System.

[225] BioMet Toolbox. http://biomet-toolbox.org/.

[226] Schellenberger J., Que R., Fleming R.M., Thiele I., Orth J.D., Feist A.M., Zielinski D.C., Bordbar A., Lewis N.E., Rahmanian S. (2011). Quantitative prediction of cellular metabolism with constraint-based models: The COBRA Toolbox v2. 0. Nat. Protoc..

[227] Hoops S., Sahle S., Gauges R., Lee C., Pahle J., Simus N., Singhal M., Xu L., Mendes P., Kummer U. (2006). COPASI–a COmplex PAthway SImulator. Bioinformatics.

[228] COPASI. http://www.copasi.org/.

[229] Shannon P., Markiel A., Ozier O., Baliga N.S., Wang J.T., Ramage D., Amin N., Schwikowski B., Ideker T. (2003). Cytoscape: A software environment for integrated models of biomolecular interaction networks. Genome Res..

[230] Saito R., Smoot M.E., Ono K., Ruscheinski J., Wang P.L., Lotia S., Pico A.R., Bader G.D., Ideker T. (2012). A travel guide to Cytoscape plugins. Nat. Methods.

[231] Boele J., Olivier B.G., Teusink B. (2012). FAME, the flux analysis and modeling environment. BMC Syst. Biol..

[232] Hoppe A., Hoffmann S., Gerasch A., Gille C., Holzhütter H.G. (2011). FASIMU: Flexible software for flux-balance computation series in large metabolic networks. BMC Bioinform..

[233] Rocha I., Maia P., Evangelista P., Vilaça P., Soares S., Pinto J.P., Nielsen J., Patil K.R., Ferreira E.C., Rocha M. (2010). OptFlux: An open-source software platform for *in silico* metabolic engineering. BMC Syst. Biol..

[234] Karp P.D., Paley S.M., Krummenacker M., Latendresse M., Dale J.M., Lee T.J., Kaipa P., Gilham F., Spaulding A., Popescu L. (2009). Pathway Tools version 13.0: Integrated software for pathway/genome informatics and systems biology. Brief Bioinform..

[235] Agren R., Liu L., Shoaie S., Vongsangnak W., Nookaew I., Nielsen J. (2013). The RAVEN toolbox and its use for generating a genome-scale metabolic model for *Penicillium chrysogenum*. PLoS Comput. Biol..

[236] Gevorgyan A., Bushell M.E., Avignone-Rossa C., Kierzek A.M. (2011). SurreyFBA: A command line tool and graphics user interface for constraint-based modeling of genome-scale metabolic reaction networks. Bioinformatics.

[237] Schellenberger J., Park J.O., Conrad T.M., Palsson B.Ø. (2010). BiGG: A Biochemical Genetic and Genomic knowledgebase of large scale metabolic reconstructions. BMC Bioinform..

[238] Caspi R., Altman T., Dale J.M., Dreher K., Fulcher C.A., Gilham F., Kaipa P., Karthikeyan A.S., Kothari A., Krummenacker M. (2010). The MetaCyc database of metabolic pathways and enzymes and the BioCyc collection of pathway/genome databases. Nucleic Acids Res..

[239] Li C., Donizelli M., Rodriguez N., Dharuri H., Endler L., Chelliah V., Li L., He E., Henry A., Stefan M.I. (2010). BioModels Database: An enhanced, curated and annotated resource for published quantitative kinetic models. BMC Syst. Biol..

[240] Schomburg I., Chang A., Placzek S., Sohngen C., Rother M., Lang M., Munaretto C., Ulas S., Stelzer M., Grote A. (2012). BRENDA in 2013: Integrated reactions, kinetic data, enzyme function data, improved disease classification: New options and contents in BRENDA. Nucleic Acids Res..

[241] Lloyd C.M., Lawson J.R., Hunter P.J., Nielsen P.F. (2008). The CellML model repository. Bioinformatics.

[242] Flicek P., Amode M.R., Barrell D., Beal K., Billis K., Brent S., Carvalho-Silva D., Clapham P., Coates G., Fitzgerald S. (2013). Ensembl 2014. Nucleic Acids Res..

[243] Artimo P., Jonnalagedda M., Arnold K., Baratin D., Csardi G., de Castro E., Duvaud S., Flegel V., Fortier A., Gasteiger E. (2012). ExPASy: SIB bioinformatics resource portal. Nucleic Acids Res..

[244] Safran M., Dalah I., Alexander J., Rosen N., Stein T.I., Shmoish M., Nativ N., Bahir I., Doniger T., Krug H. (2010). GeneCards Version 3: The human gene integrator. Database.

[245] Romero P., Wagg J., Green M.L., Kaiser D., Krummenacker M., Karp P.D. (2004). Computational prediction of human metabolic pathways from the complete human genome. Genome Biol..

[246] Olivier B.G., Snoep J.L. (2004). Web-based kinetic modelling using JWS Online. Bioinformatics.

[247] Christensen B., Nielsen J. (1999). Isotopomer analysis using GC-MS. Metab. Eng..

[248] Kelleher J.K. (2001). Flux estimation using isotopic tracers: Common ground for metabolic physiology and metabolic engineering. Metab. Eng..

[249] Kohlstedt M., Becker J., Wittmann C. (2010). Metabolic fluxes and beyond-systems biology understanding and engineering of microbial metabolism. Appl. Microbiol. Biotech..

[250] Claudino W.M., Quattrone A., Biganzoli L., Pestrin M., Bertini I., di Leo A. (2007). Metabolomics: Available results, current research projects in breast cancer, and future applications. J. Clin. Oncol..

[251] Malet-Martino M., Holzgrabe U. (2011). NMR techniques in biomedical and pharmaceutical analysis. J. Pharmaceut. Biomed..

[252] Van Q.N., Veenstra T.D. (2009). How close is the bench to the bedside? Metabolic profiling in cancer research. Genome Med..

[253] Chan S.C., Ng S.H., Chang J.T.C., Lin C.Y., Chen Y.C., Chang Y.C., Hsu C.L., Wang H.M., Liao C.T., Yen T.C. (2006). Advantages and pitfalls of 18F-fluoro-2-deoxy-D-glucose positron emission tomography in detecting locally residual or recurrent nasopharyngeal carcinoma: Comparison with magnetic resonance imaging. Eur. J. Nucl. Med. Mol. I.

[254] Chang J.M., Lee H.J., Goo J.M., Lee H.Y., Lee J.J., Chung J.K., Im J.G. (2006). False positive and false negative FDG-PET scans in various thoracic diseases. Korean J. Radiol..

[255] Antoniewicz M.R. (2013). Using multiple tracers for ^13^C metabolic flux analysis. Systems Metabolic Engineering.

[256] Metallo C.M., Walther J.L., Stephanopoulos G. (2009). Evaluation of ^13^C isotopic tracers for metabolic flux analysis in mammalian cells. J. Biotechnol..

[257] Gaglio D., Metallo C.M., Gameiro P.A., Hiller K., Sala Danna L., Balestrieri C., Alberghina L., Stephanopoulos G., Chiaradonna F. (2011). Oncogenic K-Ras decouples glucose and glutamine metabolism to support cancer cell growth. Mol. Syst. Biol..

[258] Ahn W.S., Antoniewicz M.R. (2012). Towards dynamic metabolic flux analysis in CHO cell cultures. Biotechnol. J..

[259] Antoniewicz M.R. (2013). Tandem mass spectrometry for measuring stable-isotope labeling. Curr. Opin. Biotech..

[260] Crown S.B., Antoniewicz M.R. (2013). Publishing ^13^C metabolic flux analysis studies: A review and future perspectives. Metab. Eng..

[261] Hiller K., Metallo C.M. (2013). Profiling metabolic networks to study cancer metabolism. Curr. Opin. Biotech..

[262] Metallo C.M., Gameiro P.A., Bell E.L., Mattaini K.R., Yang J., Hiller K., Jewell C.M., Johnson Z.R., Irvine D.J., Guarente L. (2012). Reductive glutamine metabolism by IDH1 mediates lipogenesis under hypoxia. Nature.

[263] Stephanopoulos G. (1999). Metabolic fluxes and metabolic engineering. Metab. Eng..

[264] Sugiura Y., Honda K., Kajimura M., Suematsu M. (2014). Visualization and quantification of cerebral metabolic fluxes of glucose in awake mice. Proteomics.

